# Supplementary material for: Do community-based singing interventions have an impact on people living with dementia and their carers? A mixed-methods study protocol
Source: BMJ Open. 2023 Nov 23;13(11):e076168. doi: 10.1136/bmjopen-2023-076168 (PMC10668143; doi:10.1136/bmjopen-2023-076168)
Supplement: Author's manuscript [file bmjopen-2023-076168.draft_revisions.pdf]

BMJ Open is committed to open peer review. As part of this commitment we make the peer review history of every article we publish publicly available.

When an article is published we post the peer reviewers' comments and the authors' responses online. We also post the versions of the paper that were used during peer review. These are the versions that the peer review comments apply to.

The versions of the paper that follow are the versions that were submitted during the peer review process. They are not the versions of record or the final published versions. They should not be cited or distributed as the published version of this manuscript.

BMJ Open is an open access journal and the full, final, typeset and author-corrected version of record of the manuscript is available on our site with no access controls, subscription charges or pay-per-view fees (http://bmjopen.bmj.com).

If you have any questions on BMJ Open's open peer review process please email info.bmjopen@bmj.com

BMJ Open

An Evaluation of Singing Intervention Services for People Living with Dementia and their Carers: A study protocol

Journal:	*BMJ Open*
Manuscript ID	bmjopen-2023-076168
Article Type:	Protocol
Date Submitted by the Author:	30-May-2023

Complete List of Authors:	Polden, Megan; University of Liverpool, Department of Primary Care & Mental Health; Lancaster University<br>Hanna, Kerry; University of Liverpool, School of health sciences<br>Ward, Kym; The Brain Charity<br>Ahmed, Faraz; Lancaster University<br>Brown, Heather; Lancaster University, Division of Health Research<br>Holland, Carol ; Lancaster University, Ageing<br>Barrow, Hazel; NIHR Applied Research Collaboration North West Coast<br>Main, Jeanette; Lyrics and Lunch Charity<br>Mann, Stella; NIHR Applied Research Collaboration North West Coast<br>Pendrill, Steve; Lyrics and Lunch Charity<br>Giebel, Clarissa; University of Liverpool, Institute of Psychology, Health and Society

Keywords:	Dementia < NEUROLOGY, Aging, MENTAL HEALTH

SCHOLARONE™
Manuscripts

An Evaluation of Singing Intervention Services for People Living with Dementia and their Carers: A study protocol

Megan Polden[1,2,5*], Kerry Hanna[3], Kym Ward[2,4], Faraz Ahmed[5], Heather Brown[5], Carol Holland[5,6], Hazel Barrow[2], Jeanette Main[2,6], Stella Mann[2], Steve Pendrill[2,6], Clarissa Giebel[1,2]

1 Department of Primary Care & Mental Health, University of Liverpool, Liverpool, UK

2 NIHR Applied Research Collaboration North West Coast, Liverpool, UK

3 School of Health Sciences, University of Liverpool, UK

4 The Brain Charity, Liverpool, UK

5 Health Research, Lancaster University, UK

6 Lyrics and Lunch Charity, UK

Corresponding author: Megan Polden, Email: m.polden@liverpool.ac.uk

Word count: 3965

For peer review only

Abstract

Background: Psychosocial Interventions have been shown to improve mood, relieve stress and improve overall quality of life for people living with dementia (PwD). To date, most evaluations of singing interventions have focused on the benefits for PwD and not their carers. This research aims to evaluate the benefits of dementia singing groups for both PwD and their carers.

Methods: This 2-year project will examine the impact of two different singing intervention services, one combining singing alongside dance and another that includes a sociable lunch. Using a mixed-methods approach, the impact of both services will be examined via the following outcome measures: quality of life, neuropsychiatric symptoms, social isolation, loneliness, cognition, carer burden and depressive symptoms in PwD and their carers. Semi-structured interviews will be conducted with a subset of (n=40) people to further examine the impact of singing services with a specific focus on barriers to access and prolonged engagement and potential for remote delivery. This project will aim to recruit n=75 PwD and their carers (n=75) in work package 1 and n=150 PwD and their carers in work package 2. A social return on investment analysis will be conducted to examine the social impact of the services.

Discussion: Increased understanding of the impact of singing interventions and how effective singing interventions are delivered will inform current and future planning for non-pharmacological interventions and support. Further understanding of the barriers that PwD encounter when accessing support services will help in making support services and interventions more widely and easily accessible.

Ethics and dissemination: Ethical approval has been obtained and all participants will provide informed consent. Results will be presented at national and international conferences and published in scientific journals and publicly disseminated to key stakeholders.

Key words: *Dementia, singing intervention, music, dance, non-pharmacological interventions, support services*

Strengths and Limitations

- This study will determine the impact of two differing and unique singing interventions for PwD and their carers using a mixed methods approach.

For peer review only - http://bmjopen.bmj.com/site/about/guidelines.xhtml

- A social return on investment analysis will be conducted to determine the social return for each singing support service.
- A comprehensive set of core outcomes will be measured in PwD and their carers
- This study will identify barriers and facilitators to accessing and engaging with singing support services after a diagnosis of dementia.
- A limitation of this study is that as participants cannot be blinded, self-report measures may include an element of bias in the responses.

For peer review only

For peer review only - http://bmjopen.bmj.com/site/about/guidelines.xhtml

1. Background

Dementia currently affects more than 55 million people worldwide[1]. Dementia has detrimental effects on cognitive functioning[2] and often leads to behavioural and psychological changes, including aggression, anxiety, hallucinations and culturally inappropriate behaviours[3-5]. Dementia has profound effects on people's quality of life and ability to perform everyday tasks[6-8]. Pharmacological interventions can aid in the management of cognitive symptoms for PwD[9]. However, the benefits are often short lived and are limited for behavioural symptoms such as anxiety, agitation and depressive symptoms[10]. Pharmacological approaches to manage behavioural symptoms in PwD include antipsychotics, anxiolytics, hypnotics and antidepressant medications[11]. There is often negative side-effect associated with using these medications including increased occurrence of strokes[12] and mortality[13,14]. Research has also suggested that antipsychotics may worsen quality of life and cognition[15-17]. The negative side effects of pharmacological interventions highlights the need for non-pharmacological interventions to reduce and help manage behavioural symptoms of dementia[18].

As a result, there is a strong need for non-pharmacological interventions such as music therapy and singing interventions[19-21]. Music therapy and singing interventions have been shown to improve mood, regulate emotion and relieve stress in older adults[22] and in PwD [23-25]. The act of singing combines language, music and instinctive human behaviour to enhance neurological stimulation[26]. Research has linked singing to improved memory performance[27] and increased cognitive functioning as it utilises and engages brain pathways other than in plain speech, which is beneficial for people with advanced stages of dementia[28-29]. Group music interventions may also help to improve social interactions between PwD, promoting relaxation and reducing levels of agitation[30,31].

It should be noted that there is mixed evidence within the literature with a systematic review of music therapy for PwD finding that music listening had a greater effect on PwD compared with singing interventions[19]. However, in recent years, research has focused on specific aspects of music therapy which result in the best outcomes for PwD[32-34]. A systematic review of multiple variations of music therapy (ranging from passive music

For peer review only - http://bmjopen.bmj.com/site/about/guidelines.xhtml

listening to active singing sessions) for PwD in residential care identified singing as an important aspect for change and improvements in mood and reductions in behavioural disturbances[35]. Further evidence from a randomised control trial suggests that singing interventions which actively engage participants may have greater effects on mood and quality of life when compared with more passive music therapy listening groups[32,36]. These studies indicate that singing and active engagement in the music sessions may be a key component for significant improvements for PwD. It is possible that this variation in findings is due to variations in dementia severity with some studies suggesting that passive music therapy may be more beneficial in more advanced stages of dementia and more active music therapy involving singing more beneficial in the early to moderate stages[37].

Research has not examined the effects of combining both singing and dancing. It is possible that combining singing and dancing would lead to increased benefits for PwD and their carers. Research examining dancing sessions in PwD has found that dancing can increase playfulness and sociability in PwD[38], improve levels of agitation and cognitive[39] and also improve physical function performance such as balance and walking speed[40]. However, more research is needed to determine to the full impacts and benefits of dancing interventions for PwD and their carers[41]. Additionally, to our knowledge, no evaluations have examined the benefits of singing interventions combined with an eating-together element. Existing literature often attributes the benefits from singing interventions directly to the singing element[42], however it may be the social aspects of the sessions which lead to the benefits for PwD and their carers[43,44]. Combining a sociable lunch alongside the singing intervention may lead to more increased and prolonged benefits of the service.

There are multiple barriers that may prevent PwD from accessing and continuing to engage with an intervention such as cost of travel, increased reliance on paid and unpaid carers and cost of service and care[45,46]. No studies to date have examined the potential barriers to accessing singing intervention services and specifically how these may differ based on social-economic position and geographical location. Accessing services after a diagnosis can be difficult[47] so understanding the benefits of a specific form of support and potential barriers to accessing these services is important.

The majority of PwD live at home where they are often cared for and supported by their friends and family[48]. Family carers are a strong factor leading to more positive outcomes

For peer review only - http://bmjopen.bmj.com/site/about/guidelines.xhtml

for PwD and can reduce or delay the need of full-time residential care[49]. It can be hugely rewarding and fulfilling caring for a family member living with dementia[50], however, it can also be challenging and have negative impacts on carers mental and physical health and quality of life[49]. Due to this, interventions such as music therapy and mindfulness have been developed to promote well-being among family and unpaid carers[51].

Singing interventions have been found to improve the relationship between PwD and their carers in addition to easing carer burden when attended together[52]. The challenges of dementia are often not limited to the PwD, with relatives, carers and friends often profoundly impacted[53]. Relatives and carers often experience increased responsibilities, stress and emotional distress as dementia progresses[54]. Due to this, there is a need for services that offer support not only for PwD but also their carers and relatives[55,56].

The aim of this 2-year project is to is to examine the benefits of dementia singing groups for both people living with dementia and their unpaid carers using both quantitative and qualitative methods. For this, the following interlinked research questions will be addressed: (1) ”Do singing interventions combined with a social lunch have an impact on well-being in PwD and their carers?”; (2) “Do singing and dancing interventions services have an impact on well-being in PwD and their carers?”; (3) “What barriers are encountered when accessing singing intervention services and do these barriers differ based on socioeconomic position (SEP)?”; (4) “Could remote delivery of singing interventions improve access and what barriers may there be with remote delivery of singing interventions?”; (5) “What is the social return on investment of the singing support services examined in this project?”.

2. Methods

2.1. Study Overview

This mixed-methods study consists of four interlinked work packages (WPs) examining the impact of singing intervention services for PwD and their carers. Mixed methods will be used to examine singing interventions provided by two registered Third Sector organisations, Lyrics and Lunch and The Brain Charity, in PwD and their carers. The barriers that people encounter when accessing and staying engaged with singing interventions

For peer review only - http://bmjopen.bmj.com/site/about/guidelines.xhtml

will be examined and whether these disproportionately affect people based on sociodemographic factors specifically SEP and location (urban vs rural location). The potential of remote delivery of singing services to help improve wider access will also be assessed. To do this, semi-structured interviews will be conducted with key stakeholders including PwD, unpaid carers, service providers (music therapists, workshop coordinators) and local facilitators (paid carers, care home managers). From this data, recommendations for future delivery of singing support services for PwD and their carers will be developed with a focus on improving access and continued engagement. A social return on investment analysis will be conducted to examined the social impact of the both the Lyrics and Lunch and The Brain Charity support services. A systematic review is also being conducted on community-based singing interventions for people living with dementia and whether they improve mood and quality of life to further inform the work (Prospero ID: CRD42023395907). This project has received ethical approval from the University of Liverpool's Ethics Committee (App ref: 12374) and Lancaster University's Ethics Committee (App ref: 3442). All methods will be performed in accordance with the relevant guidelines and regulations and all participants will provide informed consent to participate.

2.2. Two singing (and dancing) interventions

2.2.1. Intervention 1: Lyrics and Lunch

Lyrics and Lunch is a charity based in Lancaster with the aim to serve people with dementia and their carers by providing a singing group and a nourishing lunch in a sociable community environment. Lyrics and Lunch is a non-denominational Church based organisation and a short optional reflective spiritual component is included at the end, but the sessions are open to people with any religion or none. The charity currently has around 6 groups located across England. The sessions take place weekly or biweekly and last approximately 2 hours with the first hour consisting of a sociable lunch. The second hour involves group singing of familiar songs and includes percussion instruments with some of the songs. The service is open to PwD living within the community and their carers.

2.2.2. Intervention 2: The Brain Charity's Singing and Dancing Groups

For peer review only - http://bmjopen.bmj.com/site/about/guidelines.xhtml

The Brain Charity is based in Liverpool, and they support people with neurological conditions. The charity runs singing and dancing sessions in both community and residential care home settings for PwD and their carers. The singing sessions last approximately one hour and involve engaging communal singing of familiar songs such as "Happy Together" by The Turtles. The dance sessions last approximately one hour and involve physically engaging dance moves designed alongside a physical therapist and also incorporates group singing. The sessions are run weekly in 12-week blocks.

2.3. Recruitment and Sample Size

For intervention 1 (Lyrics and Lunch): PwD and carers who have accessed the Lyrics and Lunch groups or who are beginning attending will be invited to participate. A power analysis was conducted using G*Power software version 3.1.9.7. For the analysis, the power level was set at .80 with an error of .05[57]. The effect size (0.39) was based on a mean effect size taken across eight studies examining active music therapy and reported in[58]. Results revealed a minimum sample size of N= 55 is necessary to achieve a power of .80 at an alpha of .05. Therefore, to account for participant drop out and retention rates, the project will aim to recruit around n=75 PwD and n=75 of their carers from the lyrics and lunch groups.

For intervention 2 (The Brain Charity): PwD and carers who have expressed interest in accessing the Brain Charity's singing and dancing groups will be recruited prior to attending the groups. Separate samples will be collected for the singing intervention group and the dancing intervention group. Participants will be recruited from multiple singing and dancing intervention groups across the Liverpool area. These groups will include PwD living within the community and people living in residential care homes. The singing groups and dancing groups will be run by the same session facilitators.

The power analysis remained consistence with intervention 1 and revealed a minimum sample size of N= 55 is necessary to achieve a power of .80 at an alpha of .05. Therefore, across both intervention groups a total sample size of N=300 will be recruited to account for participant drop out. This will consist of n=150 PwD (n=75 from the singing group and n=75 from the dancing group) and n=150 of their carers dementia (n=75 from the singing group and n=75 from the dancing group). Data collection for the study commenced on the 25th May 2023.

For peer review only - http://bmjopen.bmj.com/site/about/guidelines.xhtml

2.4. Work Package I – Understanding the impacts of the Lyrics and Lunch support service on people with dementia and their carers

WP1 will address the first research question by examining the impacts of a singing intervention service that includes a sociable lunch aspect on PwD and their carers. This study will use quantitative methods to examine the impact of attending the singing service on cognition, social isolation, loneliness, quality of life, neuropsychiatric symptoms such as agitation or other responsive behaviours, mood (depression/anxiety) and carer burden. Additionally, the core outcome set of 13 outcomes of interventions valued by people living at home with dementia, developed directly with PwD, informal and professional carers[59] will be used. The 13 outcomes were classified into 4 domains including friendly neighbourhood and home, independence, self-managing dementia symptoms and quality of life and will be examined using a bespoke quantitative measure, an 18-item measure adapted from the Adults Social Care Outcome assessment[60], the engagement and independence in dementia questionnaire[61] and Older Americans' Resources and Services Instrumental Activities of Daily Living assessment[62]. Outcome measures will be examined when the sessions are running (ON session) and then compare this to periods of time when the session is not running such as summer holidays and Christmas breaks (OFF session) (Table 1). Participants will be required to have attended the lyrics and lunch sessions for a minimum period of 4 weeks prior to the ON session assessment. OFF session assessments will be completed after the sessions have not been attended for a minimum of 4 weeks. The Lyrics and Lunch sessions run weekly or biweekly.

2.4.1. Measures

Standard outcome measures that will be assessed in PwD and their carers will include: cognition assessed using a shortened version of the Addenbrooke's cognitive examination (ACE-III)[63] were participant's verbal fluency and attention will be assessed, quality of life assessed using the dementia quality of life measure (DemQOL and DemQOL-Proxy)[64], social Isolation assessed using the Duke Social Support Index (DSSI-10)[65], loneliness assessed using Three-item loneliness UCLA Measure[66], mood and depressive symptoms assessed using the Quick Inventory of Depressive Symptomatology (QIDS-SR)[67], neuropsychiatric

For peer review only - http://bmjopen.bmj.com/site/about/guidelines.xhtml

symptoms assessed using Neuropsychiatric Inventory Questionnaire (NPI-Q) completed by carers[68] and carer burden assessed using the Zarit burden questionnaire[69].

Demographic information will be collected from participants at the first assessment including age, gender, ethnicity, highest education level (an indicator of SEP), number of years in formal education (an indicator of SEP), years since diagnosis and years since symptom onset. Participant attendance will be recorded to determine adherence to the intervention. The Global Deterioration Scale for Assessment of Primary Degenerative Dementia will be completed by carers to determine dementia severity[70].

It is hypothesised that attendance to singing interventions will increase quality of life and mood and reduce depressive and neuropsychiatric and symptoms in PwD and their carers. Social isolation can be prominent in PwD and attending the singing intervention may reduce feelings of social isolation. Due to this, it is hypothesised that attending the singing intervention will result in reduced self-reported feelings of social isolation and loneliness in both PwD and their carers. It is hypothesised that both PwD and their carers will show improvements on the core outcome measures during the times that they are attending the singing intervention compared to period when they are not attending the sessions.

2.5. Work Package II – Understanding the impacts of singing and dancing support service conducted by The Brain Charity on people with dementia and their carers

WP2 will address the second research question and examine the impacts of a attending either a 12-week singing intervention or a 12-week dancing intervention that also involves some singing for PwD and their carers. Participants will only attend one intervention service (either the singing or the dancing intervention). The benefits of dementia singing and dancing groups for both PwD and their unpaid carers, both community-residing and those living in care homes (conducted within the care home), will be examined using a mixed-methods approach. The project will examine the impact of the services on cognition, mood, social isolation, loneliness, carer burden and neuropsychiatric symptoms.

2.5.1. Measures

For peer review only - http://bmjopen.bmj.com/site/about/guidelines.xhtml

The following outcome measures will be used: DemQol, DemQol-Proxy, QIDS-SR, ACE-III (shortened version assessing verbal fluency and attention only), NPI-Q, DSSI-10, Zarit burden questionnaire (Figure 1). Outcome measures will be examined at baseline (before the start of the 12-week block of sessions) and then one week after the final session, thus providing pre and post-intervention measures. The QIDS-SR will be completed every four weeks during the 12 weeks to examine more subtle changes in mood and depressive symptoms over the 12 weeks (Figure 1). The dance elements included in the dancing sessions may have impacts on the physical health and frailty of PwD attending the sessions. Due to this, The Physical Frailty Phenotype[71] measure of physical frailty will be included for the dancing group that will assess weight loss, grip strength, exhaustion, gait speed and physical activity. The Physical Frailty Phenotype will be completed pre and post intervention.

Similar to WP1, participant attendance will be recorded to determine adherence to the intervention. Demographic information will be collected at baseline, including age, gender, ethnicity, highest education level (an indicator of SEP), number of years in formal education (an indicator of SEP), years since diagnosis and years since symptom onset. The Global Deterioration Scale for Assessment of Primary Degenerative Dementia will be completed by carers to determine dementia severity[70].

2.6. Work Package III – Understanding the acceptability, remote delivery and barriers to accessing and continued engagement with singing interventions

WP3 will address research questions 3 and 4 and will examine barriers to accessing and continued engagement with singing interventions and how these factors may differ based on SEP and location (urban vs rural location). Accessibility barriers are likely to vary across these areas and depend on people's SEP and should be examined and acknowledged when rolling out intervention programmes on a wider scale.

A series of semi-structured follow-up interviews will be conducted with a subset of PwD, unpaid carers, service providers (music therapists, workshop coordinators) and local facilitators (paid carers, care home managers). The topic guide(s) will be coproduced with the teams and four public advisers consisting of a person living with dementia, a paid carer, an unpaid carer and a music teacher (JM) who runs the sessions.

For peer review only - http://bmjopen.bmj.com/site/about/guidelines.xhtml

The first aim will focus on barriers to access and continued engagement with the intervention service. To address this, a subset of 20 people will be recruited (approx. 10 PwD and 10 unpaid carers) from both interventions, resulting in a total of 40 interviews. This initial sample size determination was guided by information power methods[72]. These interviews will focus on the following topics: acceptability of the services, impacts of the service on mood and overall quality of life and accessibility including barriers to access and continued engagement.

Our second aim is to examine the potential of services to be adapted for remote delivery and potential barriers. When discussing remote access, adaptations made to the services when COVID-19 restrictions were in place will be discusses and how successful these remote access adaptations were. To address this, 20 people will be recruited, approx. 10 service providers (music therapists, workshop coordinators) and 10 local facilitators (paid carers, care home managers). These interviews will focus on the following topics: impacts of the service on PwD and their carers, and barriers to remote delivery of singing. Again, this initial sample size determination was guided by information power methods[72]. Preliminary analysis will be used to assess power of the analysis during the data collection phase to determine if the sample size calculation is correct. After the first three interviews, a first review of the data will be conducted, and first suggestions of relevant theory/themes will be made. This assessment will be considered again before closing data collection. Data will be analysed using Braun & Clarke's reflexive thematic analysis[73], and public advisors will assist with coding the transcripts.

2.7. Work Package IV – Examination of the social impact of singing interventions

It is important to quantify the value of singing interventions, not just in economic terms, but also in terms of social value, so in WP4, research question 5 will be addressed and the social impact of singing interventions examined by conducting a social return on investment (SROI) analysis on the Lyrics and Lunch service and on the singing and dancing services run by The Brain Charity.

Quantitative data collected for WPs I and II on the effects of the intervention on quality of life, mood, carer burden, depressive symptoms and agitation levels will be used as

For peer review only - http://bmjopen.bmj.com/site/about/guidelines.xhtml

outcome measures for the SROI analysis. For PwD living in the community, data will be collected on years since diagnosis and compare this to existing literature on average time from dementia diagnosis to care home admission to assess whether attendance to singing support services may contribute to delayed admission to a residential care home. Data on investment and running costs of the services will be provided by the Lyrics and Lunch Charity and The Brain Charity. Data will be collected on the cost for people to attend the programmes to also examine a societal perspective. Separate SROI analyses will be conducted for the Lyrics and Lunch Charity and the Brain Charity. The methods and procedure employed for both analyses will remain consistent.

Key stakeholders and people likely to be impacted by the services such as PwD, relatives, unpaid and paid carers, Lyrics and lunch and the Brain charity will first be identified. Following this the outcomes will be mapped which will be determined by interviews and engagement with identified stakeholders creating an impact map. This impact map will show the relationship between inputs, outputs and outcomes for the stakeholders. After collecting evidence on the outcomes, a monetised value will be provided to each of the outcomes including those that do not have a price attached to them such as changes in quality of life. For outcome measures that do not have a clear monitory value, established SROI value banks will be used to identify suitable financial proxies.

Percentage values will then be determined for attribution which considers changes or outcomes declared in the analysis that may not be due to just the singing intervention but may be due to other support services. A percentage value for the deadweight will be determined which considers how much change in the outcome values would have happened regardless of the singing intervention programme. Deadweight will be calculated as a 10% reduction in overall value for every resource participants identify as an alternative to the singing support service, for example if participants attend other support groups. These figures will be included in the SROI analysis[74]. To calculate the SROI the benefits will be added up and any negatives (attribution and deadweight) subtracted, and this will be compared to the investment cost of the service. This calculation will provide us with a SROI ratio which demonstrates the social value of the service in relation to the cost invested, for example an SROI ratio of £3.50: £1 means that for every pound invested there is £3.50 of social value created. Using this data, the findings will be disseminated and recommendations created for future delivery of the service.

For peer review only - http://bmjopen.bmj.com/site/about/guidelines.xhtml

2.8. Patient and Public Involvement

This project has been developed and conceptualised alongside members of the public and key stakeholders. Our formal project team includes four public advisors including a person living with dementia (SM), two carers (one paid (SP) and one unpaid (HB)) and also a music teacher (JM) who runs the singing sessions. All public advisors have previous experience and involvement with the singing sessions provided by The Brain Charity or the Lyrics and Lunch Charity. Public advisors contribute to all aspects of this research project, from designing study documents and advising on outcome measures to the interpretation of findings and dissemination of the research. Public advisors are reimbursed for the time they contribute to the project.

3.0. Discussion

Using a mixed-methods design, this project seeks to explore the impact of two established singing intervention services for PwD and their carers. The study aims to collect a large sample of key stakeholders to examine the impact of singing support services on well-being and quality of life through a combination of quantitative and qualitative methods. The findings from this research will highlight the impact of singing support services for PwD and their carers and will allow the SROI of such services to be evaluated. The project will examine the barriers that people encounter when accessing and continuing to engage with singing interventions and whether these barriers disproportionately affect people from lower SEP. It can be challenging to access support services after a diagnosis of dementia[47] and exploring ways to make services more widely and easily accessible is important. Methods to reduce potential barriers to access such as travel, increase reliance on carers and availability of services will be explored alongside the potential for remote delivery of singing services to improve and expand access. If the results from the current study suggest that singing support services have a positive impact on PwD and their carers, then support services could be scaled up and applied to other regions and settings to improve access to beneficial support services. Examining the SROI will provide an insight into the cost effectiveness of the support services and if they will be suitable to apply in lower income areas and regions both nationally and internationally. The findings from this project will be used to develop recommendations for future delivery of singing intervention services.

For peer review only - http://bmjopen.bmj.com/site/about/guidelines.xhtml

Abbreviation List

PwD	People living with dementia
WP	Work package
SEP	Socioeconomic Position
DemQOL	Dementia quality of life measure
ACE-III	Addenbrooke's cognitive examination
DSSI-10	Duke Social Support Index
QIDS-SR	Quick Inventory of Depressive Symptomatology
NPI-Q	Neuropsychiatric Inventory Questionnaire
SROI	Social return on investment

Ethics Approval and Consent to Participate: This project has received ethical approval from the University of Liverpool's Ethics Committee (App ref: 12374) and Lancaster University's Ethics Committee (App ref: 3442). All methods will be performed in accordance with the relevant guidelines and regulations and all participants will provide informed consent to participate.

Conflicts of Interest: Carol Holland, Jeanette Main and Steve Pendrill are trustees of the Lyrics and Lunch Charity. All other authors declare no conflict of interest.

Consent for publication: N/A

Funding: This is independent research funded jointly by the National Institute for Health and Care Research Applied Research Collaboration North West Coast (ARC NWC) and The Alzheimer's Society. The views expressed in this publication are those of the author(s) and not necessarily those of the National Institute for Health Research or the Department of Health and Social Care or The Alzheimer's Society.

Author Contributions: Funding was obtained by MP, KH, KW, FA, HB, CH and CG. Conceptualisation and methodology design contributions and project administration contributions were made by all authors. The original draft preparation contributions were made by MP. All authors contributed to revising the manuscript. All authors read and agreed to the published version of the manuscript.

For peer review only - http://bmjopen.bmj.com/site/about/guidelines.xhtml

Availability of data and materials: Data generated in this study will be made available on Open Science Framework upon completion of the study.

Acknowledgements: N/A

References

1. World Health Organization . Geneva, Switzerland: World Health Organization; 2021. Fact sheets of dementia [Internet]. Available from: https:// www.who.int/news-room/fact-sheets/detail/dementia
2. Sliwinski M, Lipton RB, Buschke H, Stewart W. The effects of preclinical dementia on estimates of normal cognitive functioning in aging. The Journals of Gerontology Series B: Psychological Sciences and Social Sciences. 1996 Jul 1;51(4):P217-25.
3. Srikanth S, Nagaraja AV, Ratnavalli E. Neuropsychiatric symptoms in dementia-frequency, relationship to dementia severity and comparison in Alzheimer's disease, vascular dementia and frontotemporal dementia. Journal of the neurological sciences. 2005 Sep 15;236(1-2):43-8.
4. Cagnin A, Gnoato F, Jelcic N, Favaretto S, Zarantonello G, Ermani M, Dam M. Clinical and cognitive correlates of visual hallucinations in dementia with Lewy bodies. Journal of Neurology, Neurosurgery & Psychiatry. 2013 May 1;84(5):505-10.
5. Kwak YT, Yang Y, Koo MS. Anxiety in dementia. Dementia and neurocognitive disorders. 2017 Jun;16(2):33.
6. Banerjee S, Smith SC, Lamping DL, Harwood RH, Foley B, Smith P, Murray J, Prince M, Levin E, Mann A, Knapp M. Quality of life in dementia: more than just cognition. An analysis of associations with quality of life in dementia. Journal of Neurology, Neurosurgery & Psychiatry. 2006 Feb 1;77(2):146-8.
7. Giebel CM, Sutcliffe C, Challis D. Hierarchical decline of the initiative and performance of complex activities of daily living in dementia. Journal of geriatric psychiatry and neurology. 2017;30(2):96-103.
8. Resnick B, Galik E, Kolanowski A, VanHaitsma K, Boltz M, Zhu S, Ellis J, Behrens L, Eshraghi K, Renn C, Dorsey SG. The relationship between pain, function, behavioral, and psychological symptoms of dementia and quality of life. Pain Management Nursing. 2022 Feb 1;23(1):55-61.
9. Watts KE, Storr NJ, Barr PG, Rajkumar AP. Systematic review of pharmacological interventions for people with Lewy body dementia. Aging & Mental Health. 2023 Feb 1;27(2):203-16.
10. Gerlach LB, Kales HC. Pharmacological management of neuropsychiatric symptoms of dementia. Current treatment options in psychiatry. 2020 Dec;7:489-507.
11. Gustafsson M, Karlsson S, Gustafson Y, Lövheim H. Psychotropic drug use among people with dementia–a six-month follow-up study. BMC Pharmacology and Toxicology. 2013 Dec;14(1):1-9.
12. Maidment ID, Damery S, Campbell N, Seare N, Fox C, Iliffe S, Hilton A, Brown G, Barnes N, Wilcock J, Randle E. Medication review plus person-centred care: a feasibility study of a pharmacy-health psychology dual intervention to improve care for people living with dementia. BMC psychiatry. 2018 Dec;18(1):1-1.
13. Maust DT, Kim HM, Seyfried LS, Chiang C, Kavanagh J, Schneider LS, Kales HC. Antipsychotics, other psychotropics, and the risk of death in patients with dementia: number needed to harm. JAMA psychiatry. 2015 May 1;72(5):438-45.

For peer review only - http://bmjopen.bmj.com/site/about/guidelines.xhtml

14. Tampi RR, Tampi DJ, Balachandran S, Srinivasan S. Antipsychotic use in dementia: a systematic review of benefits and risks from meta-analyses. Therapeutic advances in chronic disease. 2016 Sep;7(5):229-45.
15. Ballard C, Corbett A. Management of neuropsychiatric symptoms in people with dementia. CNS drugs. 2010 Sep;24:729-39.
16. Maidment ID, Shaw RL, Killick K, Damery S, Hilton A, Wilcock J, Barnes N, Brown G, Gillespie S, Fox C, Barton G. Improving the management of behaviour that challenges associated with dementia in care homes: protocol for pharmacy–health psychology intervention feasibility study. BMJ open. 2016 Mar 1;6(3):e010279.
17. Rochon PA, Normand SL, Gomes T, Gill SS, Anderson GM, Melo M, Sykora K, Lipscombe L, Bell CM, Gurwitz JH. Antipsychotic therapy and short-term serious events in older adults with dementia. Archives of internal medicine. 2008 May 26;168(10):1090-6.
18. O'Donnell E, Holland C, Swarbrick C. Strategies used by care home staff to manage behaviour that challenges in dementia: A systematic review of qualitative studies. International Journal of Nursing Studies. 2022 Apr 22:104260.
19. Moreno-Morales C, Calero R, Moreno-Morales P, Pintado C. Music therapy in the treatment of dementia: A systematic review and meta-analysis. Frontiers in medicine. 2020 May 19;7:160.
20. Dimitriou TD, Verykouki E, Papatriantafyllou J, Konsta A, Kazis D, Tsolaki M. Non-Pharmacological interventions for the anxiety in patients with dementia. A cross-over randomised controlled trial. Behavioural brain research. 2020 Jul 15;390:112617.
21. Emblad SY, Mukaetova-Ladinska EB. Creative art therapy as a non-pharmacological intervention for dementia: A systematic review. Journal of Alzheimer's Disease Reports. 2021 Jan 1;5(1):353-64.
22. Clair AA, Memmott J. Therapeutic uses of music with older adults. American Music Therapy Association. 8455 Colesville Road Suite 1000, Silver Spring, MD 20910; 2008.
23. Davidson JW, Almeida RA. An exploratory study of the impact of group singing activities on lucidity, energy, focus, mood and relaxation for persons with dementia and their caregivers. Psychology of Well-Being. 2014 Dec;4(1):1-3.
24. Lee S, O'Neill D, Moss H. Promoting well-being among people with early-stage dementia and their family carers through community-based group singing: a phenomenological study. Arts & Health. 2022a Jan 2;14(1):85-101.
25. Lee S, Allison T, O'Neill D, Punch P, Helitzer E, Moss H. Integrative review of singing and music interventions for family carers of people living with dementia. Health Promotion International. 2022b May;37(Supplement_1):i49-61.
26. Jeffries, K. J., Fritz, J. B., & Braun, A. R. (2003). Words in melody: an H215O PET study of brain activation during singing and speaking. *Neuroreport*, *14*(5), 749-754.
27. Carruth EK. The effects of singing and the spaced retrieval technique on improving face-name recognition in nursing home residents with memory loss. Journal of Music Therapy. 1997 Oct 1;34(3):165-86.
28. Satoh M, Yuba T, Tabei KI, Okubo Y, Kida H, Sakuma H, Tomimoto H. Music therapy using singing training improves psychomotor speed in patients with Alzheimer's disease: a neuropsychological and fMRI study. Dementia and geriatric cognitive disorders extra. 2015;5(3):296-308.
29. Mansens D, Deeg DJ, Comijs HC. The association between singing and/or playing a musical instrument and cognitive functions in older adults. Aging & Mental Health. 2018 Aug 3;22(8):970-7.
30. Lin Y, Chu H, Yang CY, Chen CH, Chen SG, Chang HJ, Hsieh CJ, Chou KR. Effectiveness of group music intervention against agitated behavior in elderly persons with dementia. International journal of geriatric psychiatry. 2011 Jul;26(7):670-8.
31. Unadkat S, Camic PM, Vella-Burrows T. Understanding the experience of group singing for couples where one partner has a diagnosis of dementia. The Gerontologist. 2017 Jun 1;57(3):469-78.

For peer review only - http://bmjopen.bmj.com/site/about/guidelines.xhtml

32. Li HC, Wang HH, Chou FH, Chen KM. The effect of music therapy on cognitive functioning among older adults: A systematic review and meta-analysis. Journal of the American Medical Directors Association. 2015 Jan 1;16(1):71-7.
33. Lam HL, Li WT, Laher I, Wong RY. Effects of music therapy on patients with dementia—A systematic review. Geriatrics. 2020 Sep 25;5(4):62.
34. Dowson B, Schneider J. Online singing groups for people with dementia: scoping review. Public Health. 2021 May 1;194:196-201.
35. McDermott O, Crellin N, Ridder HM, Orrell M. Music therapy in dementia: a narrative synthesis systematic review. International journal of geriatric psychiatry. 2013 Aug;28(8):781-94.
36. Cho HK. The effects of music therapy-singing group on quality of life and affect of persons with dementia: a randomized controlled trial. Frontiers in medicine. 2018 Oct 15;5:279.
37. Baker FA, Lee YE, Sousa TV, Stretton-Smith PA, Tamplin J, Sveinsdottir V, Geretsegger M, Wake JD, Assmus J, Gold C. Clinical effectiveness of music interventions for dementia and depression in elderly care (MIDDEL): Australian cohort of an international pragmatic cluster-randomised controlled trial. The Lancet Healthy Longevity. 2022 Mar 1;3(3):e153-65.
38. Kontos P, Grigorovich A, Kosurko A, Bar RJ, Herron RV, Menec VH, Skinner MW. Dancing with dementia: Exploring the embodied dimensions of creativity and social engagement. The Gerontologist. 2021 Aug;61(5):714-23.
39. Salihu D, Wong EM, Bello UM, Kwan RY. Effects of dance intervention on agitation and cognitive functioning of people living with dementia in institutional care facilities: Systematic review. Geriatric Nursing. 2021 Nov 1;42(6):1332-40.
40. Bracco L, Poirier G, Pinto-Carral A, Mourey F. Effect of Dance Therapy on the Physical Abilities of Older Adults with Dementia: A Systematic Review. Eur J Geriatr Gerontol. 2021 Dec 1;3(3):134-42.
41. Karkou V, Meekums B. Dance movement therapy for dementia. Cochrane Database of Systematic Reviews. 2017(2).
42. Adkins-Regan E. Activity dependent brain plasticity: does singing increase the volume of a song system nucleus? Theoretical comment on Sartor and Ball (2005).
43. Galinha I, Pinal D, Lima ML, Labisa-Palmeira A. The role of social and physiological variables on older adults' cognitive improvement after a group singing intervention: the Sing4health randomized controlled trial. Psychosocial Intervention. 2021;30(3):123-38.
44. Clark IN, Tamplin JD, Baker FA. Community-dwelling people living with dementia and their family caregivers experience enhanced relationships and feelings of well-being following therapeutic group singing: A qualitative thematic analysis. Frontiers in Psychology. 2018 Jul 30;9:1332.
45. Morgan DG, Semchuk KM, Stewart NJ, D'arcy C. Rural families caring for a relative with dementia: Barriers to use of formal services. Social science & medicine. 2002 Oct 1;55(7):1129-42.
46. Hossain MZ, Khan HT. Barriers to access and ways to improve dementia services for a minority ethnic group in England. Journal of evaluation in clinical practice. 2020 Dec;26(6):1629-37.
47. Giebel C, Robertson S, Beaulen A, Zwakhalen S, Allen D, Verbeek H. "Nobody Seems to Know Where to Even Turn To": Barriers in Accessing and Utilising Dementia Care Services in England and The Netherlands. International Journal of Environmental Research and Public Health. 2021 Nov 22;18(22):12233.
48. Dickinson C, Dow J, Gibson G, Hayes L, Robalino S, Robinson L. Psychosocial intervention for carers of people with dementia: what components are most effective and when? A systematic review of systematic reviews. International psychogeriatrics. 2017 Jan;29(1):31-43.
49. Farina N, Page TE, Daley S, Brown A, Bowling A, Basset T, Livingston G, Knapp M, Murray J, Banerjee S. Factors associated with the quality of life of family carers of people with dementia: A systematic review. Alzheimer's & Dementia. 2017 May 1;13(5):572-81.

For peer review only - http://bmjopen.bmj.com/site/about/guidelines.xhtml

50. Laver K, Milte R, Dyer S, Crotty M. A systematic review and meta-analysis comparing carer focused and dyadic multicomponent interventions for carers of people with dementia. Journal of Aging and Health. 2017 Dec;29(8):1308-49.
51. Teahan A, Lafferty A, McAuliffe E, Phelan A, O'Sullivan L, O'Shea D, Nicholson E, Fealy G. Psychosocial interventions for family carers of people with dementia: A systematic review and meta-analysis. Journal of Aging and Health. 2020 Oct;32(9):1198-213.
52. Lee S, Allison T, O'Neill D, Punch P, Helitzer E, Moss H. Integrative review of singing and music interventions for family carers of people living with dementia. Health Promotion International. 2022 May;37(Supplement_1):i49-61.
53. Sutcliffe C, Giebel C, Bleijlevens M, Lethin C, Stolt M, Saks K, Soto ME, Meyer G, Zabalegui A, Chester H, Challis D. Caring for a person with dementia on the margins of long-term care: a perspective on burden from 8 European countries. Journal of the American Medical Directors Association. 2017 Nov 1;18(11):967-73.
54. Swall A, Hammar LM, Gransjön Craftman Å. Like a bridge over troubled water–a qualitative study of professional caregiver singing and music as a way to enable person-centred care for persons with dementia. International journal of qualitative studies on health and well-being. 2020 Jan 1;15(1):1735092.
55. Alwin J, Öberg B, Krevers B. Support/services among family caregivers of persons with dementia—perceived importance and services received. International Journal of Geriatric Psychiatry: A journal of the psychiatry of late life and allied sciences. 2010 Mar;25(3):240-8.
56. Bayly M, Morgan D, Chow AF, Kosteniuk J, Elliot V. Dementia-related education and support service availability, accessibility, and use in rural areas: Barriers and solutions. Canadian Journal on Aging/La Revue canadienne du vieillissement. 2020 Dec;39(4):545-85.
57. Faul F, Erdfelder E, Lang AG, Buchner A. G* Power 3: A flexible statistical power analysis program for the social, behavioral, and biomedical sciences. Behavior research methods. 2007 May;39(2):175-91.
58. Vasionytė I, Madison G. Musical intervention for patients with dementia: a meta-analysis. Journal of clinical nursing. 2013 May;22(9-10):1203-16.
59. Reilly ST, Harding AJ, Morbey H, Ahmed F, Williamson PR, Swarbrick C, Leroi I, Davies L, Reeves D, Holland F, Hann M. What is important to people with dementia living at home? A set of core outcome items for use in the evaluation of non-pharmacological community-based health and social care interventions. Age and ageing. 2020 Jul 1;49(4):664-71.
60. Johnstone L, Page C. Using Adult Social Care Outcomes Toolkit (ASCOT) in the assessment and review process. Res Policy Plann. 2013;30(3):179-92.
61. Stoner, C.R., Orrell, M., & Spector, A. (2018). Psychometric properties and factor analysis of the Engagement and Independence in Dementia Questionnaire (EID-Q). Dementia and Geriatric Cognitive Disorders, 46, 3-4, 119 – 127. DOI:10.1159/000488484
62. Doble SE, Fisher AG. The dimensionality and validity of the Older Americans Resources and Services (OARS) Activities of Daily Living (ADL) scale. Journal of Outcome Measurement. 1998 Jan 1;2(1):4-24.
63. Hsieh S, Schubert S, Hoon C, Mioshi E, Hodges JR. Validation of the Addenbrooke's Cognitive Examination III in frontotemporal dementia and Alzheimer's disease. Dementia and geriatric cognitive disorders. 2013;36(3-4):242-50.
64. Smith SC, Lamping DL, Banerjee S, Harwood R, Foley B, Smith P, Cook JC, Murray J, Prince M, Levin E, Mann A. Measurement of health-related quality of life for people with dementia: development of a new instrument (DEMQOL) and an evaluation of current methodology. Health Technology Assessment (Winchester, England). 2005;9(10):1-iv.
65. Koenig HG, Westlund RE, George LK, Hughes DC, Blazer DG, Hybels C. Abbreviating the Duke Social Support Index for use in chronically ill elderly individuals. Psychosomatics. 1993 Jan 1;34(1):61-9.
66. Russell DW. UCLA Loneliness Scale (Version 3): Reliability, validity, and factor structure. Journal of personality assessment. 1996 Feb 1;66(1):20-40.
67. Rush AJ, Trivedi MH, Ibrahim HM, Carmody TJ, Arnow B, Klein DN, Markowitz JC, Ninan PT, Kornstein S, Manber R, Thase ME. The 16-Item Quick Inventory of Depressive Symptomatology (QIDS), clinician rating (QIDS-C), and self-report (QIDS-SR): a

For peer review only - http://bmjopen.bmj.com/site/about/guidelines.xhtml

psychometric evaluation in patients with chronic major depression. Biological psychiatry. 2003 Sep 1;54(5):573-83.
68. Kaufer DI, Cummings JL, Ketchel P, Smith V, MacMillan A, Shelley T, Lopez OL, DeKosky ST. Validation of the NPI-Q, a brief clinical form of the Neuropsychiatric Inventory. The Journal of neuropsychiatry and clinical neurosciences. 2000 May;12(2):233-9.
69. Yap P. Validity and reliability of the Zarit Burden Interview in assessing caregiving burden. Ann Acad Med Singapore. 2010;39:758-63.0 May;12(2):233-9.
70. Reisberg B, Ferris SH, de Leon MJ, Crook T. Global Deterioration Scale. The American Journal of Psychiatry. 1983.
71. Fried LP, Tangen CM, Walston JD, Newman AB, Hirsch C, Gottdiener JS. E Seeman, T.; Tracy, RP; Kop, WJ; Burke, GL; et al. Frailty in older adults: Evidence for a phenotype. J. Gerontol. Ser. A Boil. Sci. Med. Sci. 2001;56:M146-57.
72. Malterud K, Siersma VD, Guassora AD. Sample size in qualitative interview studies: guided by information power. Qualitative health research. 2016 Nov;26(13):1753-60.
73. Braun, V., & Clarke, V. (2021). One size fits all? What counts as quality practice in (reflexive) thematic analysis?. *Qualitative research in psychology*, *18*(3), 328-352.
74. Then V, Schober C, Rauscher O, Kehl K. Social return on investment analysis. Social Return on Investment Analysis. https://doi. org/10.1007/978-3-319-71401-1. 2017.

Tables and Figures

Table 1. Assessments to be completed by PwD and their carers

**Assessment Time period**	**Assessment completed by Person living with Dementia**	**Assessment completed by unpaid carer**
**ON session (Participant has attended service for a minimum of 4 weeks)**	QIDS-SR, ACE, DemQOL, DSSI-10	QIDS-SR, DSSI-10, NPI-Q, DemQOL-Proxy, Zarit burden questionnaire
**OFF session (Participant has not attended a session for a minimum of 4 weeks)**	QIDS-SR, ACE, DemQOL, DSSI-10	QIDS-SR, DSSI-10, NPI-Q, DemQOL-Proxy, Zarit burden questionnaire

For peer review only - http://bmjopen.bmj.com/site/about/guidelines.xhtml

Figure 1. Timeline of cognitive assessments for PwD and unpaid carers

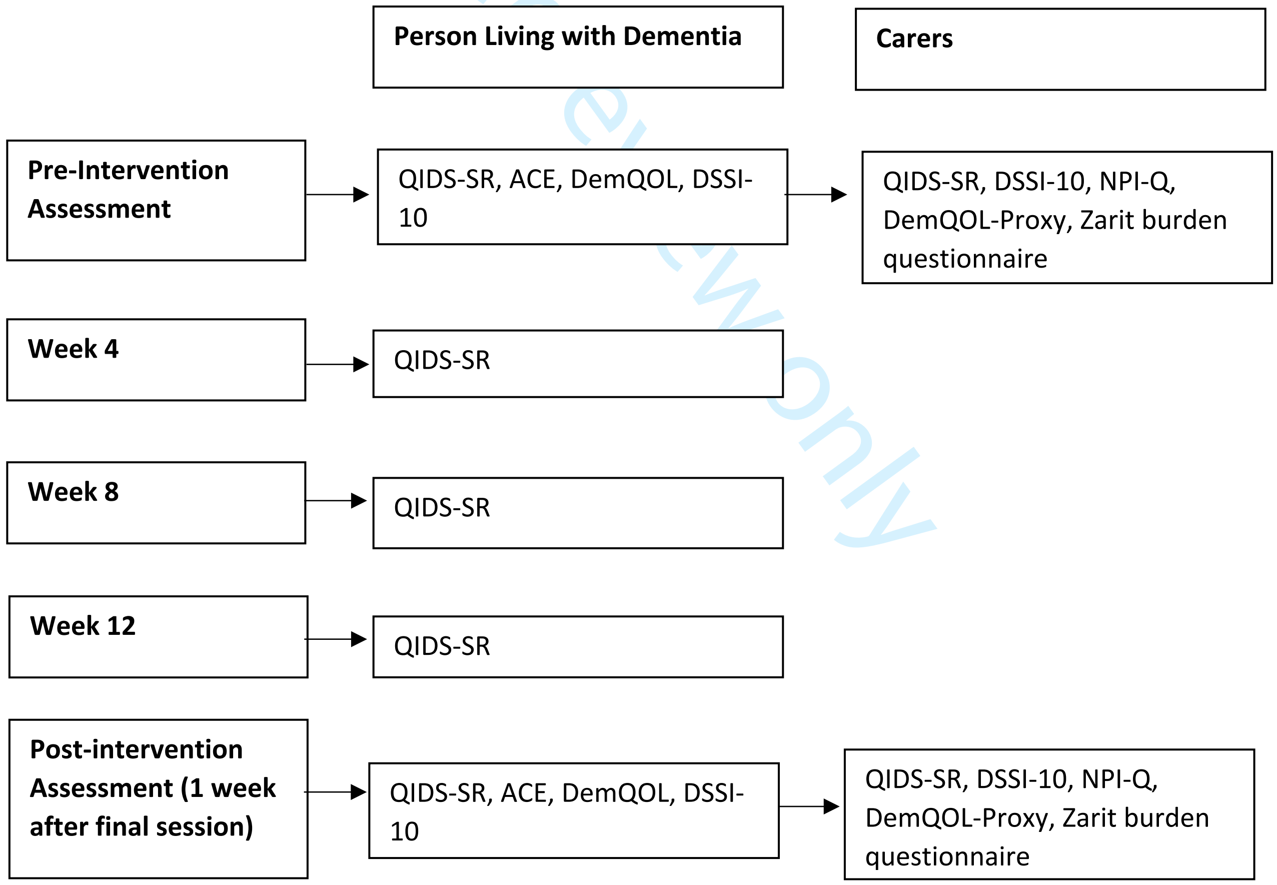

For peer review only

BMJ Open

Do community-based singing interventions have an impact on people living with dementia and their carers? A mixed-methods study protocol

Journal:	*BMJ Open*
Manuscript ID	bmjopen-2023-076168.R1
Article Type:	Protocol
Date Submitted by the Author:	05-Oct-2023
Complete List of Authors:	Polden, Megan; University of Liverpool, Department of Primary Care & Mental Health; Lancaster University<br>Hanna, Kerry; University of Liverpool, School of health sciences<br>Ward, Kym; The Brain Charity<br>Ahmed, Faraz; Lancaster University<br>Brown, Heather; Lancaster University, Division of Health Research<br>Holland, Carol ; Lancaster University, Ageing<br>Barrow, Hazel; NIHR Applied Research Collaboration North West Coast<br>Main, Jeanette; Lyrics and Lunch Charity<br>Mann, Stella; NIHR Applied Research Collaboration North West Coast<br>Pendrill, Steve; Lyrics and Lunch Charity<br>Giebel, Clarissa; University of Liverpool, Institute of Psychology, Health and Society
**Primary Subject Heading**:	Sociology
Secondary Subject Heading:	Mental health, Sociology, Public health
Keywords:	Dementia < NEUROLOGY, Aging, MENTAL HEALTH

SCHOLARONE™
Manuscripts

Do community-based singing interventions have an impact on people living with dementia and their carers? A mixed-methods study protocol

Megan Polden[1,2,5*], Kerry Hanna[3], Kym Ward[2,4], Faraz Ahmed[5], Heather Brown[5], Carol Holland[5,6], Hazel Barrow[2], Jeanette Main[2,6], Stella Mann[2], Steve Pendrill[2,6], Clarissa Giebel[1,2]

1 Department of Primary Care & Mental Health, University of Liverpool, Liverpool, UK

2 NIHR Applied Research Collaboration North West Coast, Liverpool, UK

3 School of Health Sciences, University of Liverpool, UK

4 The Brain Charity, Liverpool, UK

5 Health Research, Lancaster University, UK

6 Lyrics and Lunch Charity, UK

*Corresponding author: Megan Polden, Email: m.polden@liverpool.ac.uk

For peer review only

Word count: 5024

For peer review only - http://bmjopen.bmj.com/site/about/guidelines.xhtml

Abstract

Introduction: Psychosocial interventions have been shown to improve mood, relieve stress and improve quality of life for people living with dementia (PwD). To date, most evaluations of singing interventions have focused on the benefits for PwD and not their carers. This research aims to evaluate the benefits of dementia singing groups for both PwD and their carers.

Methods and Analysis: This 2-year project will observe the impact of two different singing intervention services, one combining singing alongside dance and another that includes a sociable lunch. This project will aim to recruit a total of n=150 PwD and n=150 carers across the two singing interventions. Using a mixed-methods approach, the influence of both services will be analysed via the following outcome measures: quality of life, neuropsychiatric symptoms, social isolation, loneliness, cognition, carer burden and depressive symptoms in PwD and their carers using a pre/post study design. Regression models will be used to analyse the data with time (pre/post) as the exposure variable. Semi-structured interviews will be conducted with a subset of (n=40) people to further the impact of singing services with a specific focus on acceptability of the interventions, barriers to access and prolonged engagement and potential for remote delivery. Interview data will be analysed using Braun & Clarke's reflexive thematic analysis, and public advisors will assist with coding the transcripts. A social return on investment analysis will be conducted to determine the social impact of the services.

Discussion: Increased understanding of the influence of singing interventions and how effective singing interventions are delivered will inform current and future planning for non-pharmacological interventions and support. Further understanding of the barriers that PwD encounter when accessing support services will help in making support services and interventions more widely and easily accessible.

Ethics and dissemination: This project has received ethical approval from the University of Liverpool's Ethics Committee (App ref: 12374) and Lancaster University's Ethics Committee (App ref: 3442). All participants will provide informed consent to participate. Results will be presented at national and international conferences and published in scientific journals and publicly disseminated to key stakeholders.

For peer review only - http://bmjopen.bmj.com/site/about/guidelines.xhtml

Key words: *Dementia, singing intervention, music, dance, non-pharmacological interventions, support services*

Strengths and Limitations

- This study will determine the impact of two differing and unique singing interventions for PwD and their carers using a mixed methods approach.
- A social return on investment analysis will be conducted to determine the social return for each singing support service.
- A comprehensive set of core outcomes will be measured in PwD and their carers
- This study will identify barriers and facilitators to accessing and engaging with singing support services after a diagnosis of dementia.
- A limitation of this study is that as participants cannot be blinded, self-report measures may include an element of bias in the responses.

For peer review only - http://bmjopen.bmj.com/site/about/guidelines.xhtml

1. Introduction

Dementia currently affects more than 55 million people worldwide [1]. Dementia has detrimental effects on cognitive functioning [2] and often leads to behavioural and psychological changes, including aggression, anxiety, hallucinations and culturally inappropriate behaviours [3-5]. Dementia has profound effects on people's quality of life and ability to perform everyday tasks [6-8]. Pharmacological interventions can aid in the management of cognitive symptoms for PwD [9]. However, the benefits are often short lived and are limited for behavioural symptoms such as anxiety, agitation and depressive symptoms [10]. Pharmacological approaches to manage behavioural symptoms in PwD include antipsychotics, anxiolytics, hypnotics and antidepressant medications [11]. There is often negative side-effect associated with using these medications including increased occurrence of strokes [12] and mortality [13,14]. Research has also suggested that antipsychotics may worsen quality of life and cognition [15-17]. The negative side effects of pharmacological interventions highlights the need for non-pharmacological interventions to reduce and help manage behavioural symptoms of dementia [18].

As a result, there is a strong need for non-pharmacological interventions such as music therapy which has demonstrated strong benefits for PwD [19,20]. Music therapies have been shown to improve mood, regulate emotion and relieve stress in older adults [21] and in PwD [22-24]. Music therapy as a whole consists of many components and applications and one specific and promising form of music therapy is singing interventions. The act of singing combines language, music and instinctive human behaviour to enhance neurological stimulation [25]. Research has linked singing to improved memory performance [26] and increased cognitive functioning as it utilises and engages brain pathways other than in plain speech, which is beneficial for people with advanced stages of dementia [27,28]. Group singing interventions may also help to improve social interactions between PwD, promoting relaxation and reducing levels of agitation [29,30]. Singing interventions in particular have demonstrated value and effectiveness within the realm of music therapies.

It should be noted that there is mixed evidence within the literature with a systematic review of music therapy for PwD finding that music listening had a greater effect on PwD compared with singing interventions [19]. However, in recent years, research has focused on specific aspects of music therapy which result in the best outcomes for PwD [31-33]. A systematic review of multiple variations of music therapy (ranging from passive music listening to active singing sessions) for PwD in residential care identified singing as an important aspect for change and improvements in mood and reductions in behavioural

For peer review only - http://bmjopen.bmj.com/site/about/guidelines.xhtml

disturbances [34]. Further evidence from a randomised control trial suggests that singing interventions which actively engage participants may have greater effects on mood and quality of life when compared with passive music therapy [31,35]. These studies indicate that singing and active engagement in the music sessions may be a key component for significant improvements for PwD. It is possible that this variation in findings is due to variations in dementia severity with some studies suggesting that passive music therapy may be more beneficial in more advanced stages of dementia and more active music therapy involving singing more beneficial in the early to moderate stages [36].

PwD can often experience a decrease in physical activity levels post diagnosis which can lead to adverse effects on physical health such as reductions in strength, balance, mobility and increased risk of physical frailty. Increased physical activity has been associated with positive cognitive and physical outcomes in PwD [37] and a reduction in symptom progression. Review evidence has found that multidomain interventions may be more beneficial for PwD [38]. Research examining dancing sessions in PwD has found that dancing can increase playfulness and sociability in PwD [39], improve levels of agitation and cognitive function [40] and also improve physical performance such as balance and walking speed [41]. Interventions that combine physical aspects alongside cognitive stimulation may lead to increased benefits and a wider range of positive outcomes. Research has not examined the effects of combining both singing and dancing. It is possible that and intervention combining both physical (dancing) and cognitive (singing) elements could lead to increased benefits for PwD and their carers.

Research has found that people with limited social networks and reduced social engagement may be more at risk of developing dementia compared to those with wider and richer social networks [42]. It is thought that social activity and engagement may be a protective factor against cognitive decline by increasing cognitive reserve to better maintain cognitive functioning and performance [42]. To our knowledge, no evaluations have examined the benefits of singing interventions combined with an eating-together element which provides a sociable experience alongside the singing session. Existing literature often attributes the benefits from singing interventions directly to the singing element [43], however it may be the social aspects of the sessions which lead to the benefits for PwD and their carers [44,45]. The current project will investigate the impacts of two established charity-based services provided by The Brain Charity and the Lyrics and Lunch charity for PwD and their carers.

For peer review only - http://bmjopen.bmj.com/site/about/guidelines.xhtml

Most PwD live at home where they are often cared for and supported by their friends and family [46]. Family carers are a strong factor leading to more positive outcomes for PwD and can reduce or delay the need of residential care [47]. It can be hugely rewarding and fulfilling caring for a family member living with dementia [48], however, it can also be challenging and have negative impacts on the carers' mental and physical health and quality of life [47]. Due to this, interventions such as music therapy have been developed to promote well-being among unpaid carers [49]. Singing interventions have been found to improve the relationship between PwD and their carers in addition to easing carer burden when attended together [50]. The challenges of dementia are often not limited to the PwD, with relatives, carers and friends often profoundly impacted [51]. Therefore, there is a need for services to offer support not only for PwD but also their carers [52,53].

There are multiple barriers that may prevent PwD from accessing and continuing to engage with an intervention such as cost of travel, increased reliance on paid and unpaid carers and cost of service and care [54,55]. Studies to date have not examined the potential barriers to accessing singing intervention services and specifically how these may differ based on social-economic position and geographical location. Accessing services after a diagnosis can be difficult [56] so understanding the benefits of a specific form of support and potential barriers to accessing these services is important. The current study examines barriers and facilitators for accessing and continuing to engage with singing intervention services and the organisations that provide these services.

Remote delivery of singing interventions services may be a solution to some barriers for attending singing interventions such as travel or reliance on carers. During the COVID-19 pandemic multiple dementia support groups were moved online and demonstrated the potential for singing interventions to be delivered remotely [57,58]. However, the feasibility, acceptability and whether remote delivery yields similar benefits as in-person groups has been questioned [57]. For people living with advanced stages of dementia barriers may prevent them attending in-person groups and in these cases remote delivery may be a method to improve access and reach of these services.

It is important to quantify the value of interventions, not just in economic terms, but also in terms of social value. Social Return on Investment (SROI) is a method of cost-benefit analysis that assigns monetary values to social outcomes that usually would not be accounted for in standard financial evaluations. SROI analysis can be used to determine an intervention's social value in relation to financial investment [59]. SROI analysis can be a useful tool for examining interventions and to inform policy-making relating to social support

For peer review only - http://bmjopen.bmj.com/site/about/guidelines.xhtml

investments [60,61]. In the current project, the SROI will be examined for both support services.

The aim of this 2-year project is to is to examine the benefits of established charity provided dementia singing groups for both people living with dementia and their unpaid carers using both quantitative and qualitative methods. For this, the following interlinked research questions will be addressed: (1) ”Do singing interventions combined with a social lunch have an impact on well-being in PwD and their carers?”; (2) “Do singing and dancing interventions services have an impact on well-being in PwD and their carers?”; (3) “What barriers are encountered when accessing singing intervention services and what prevents continued engagement?”; (4) “Could remote delivery of singing interventions improve access and what barriers may there be with remote delivery of singing interventions?”; (5) “What is the social return on investment of the singing support services examined in this project?”.

2. Methods and Analysis

2.1. Study Overview

This mixed-methods study consists of four interlinked work packages (WPs) examining the influence of singing services provided by two registered third sector organisations, Lyrics and Lunch and The Brain Charity. The barriers that people encounter when accessing and staying engaged with singing interventions will be examined and whether these disproportionately affect people based on sociodemographic factors specifically SEP and location (urban vs rural location). The potential of remote delivery of singing services to help improve wider access will also be assessed. To do this, semi-structured interviews will be conducted with key stakeholders including PwD, unpaid carers, service providers (music therapists, workshop coordinators) and local facilitators (paid carers, care home managers). From this data, recommendations for future delivery of singing support services will be developed with a focus on improving access and continued engagement. A SROI will be conducted to examined the social impact of both support services. A systematic review is also being conducted on community-based singing interventions for people living with dementia and whether they improve mood and quality of life to further inform the work (Prospero ID: CRD42023395907).

2.2. Two singing (and dancing) interventions

2.2.1. Intervention 1: Lyrics and Lunch

For peer review only - http://bmjopen.bmj.com/site/about/guidelines.xhtml

Lyrics and Lunch is a charity based in Lancaster with the aim to serve people with dementia and their carers by providing a singing group and a nourishing lunch in a sociable community environment. The sessions take place weekly or biweekly and last approximately 2 hours with the first hour consisting of a sociable lunch followed by an hour of group singing. For the singing session attendees will be provided with a book with the lyrics to each song. Each session will start with the group singing an original song that allows each person to introduce themselves. The session leader will play piano or guitar and facilitate the group to sing along. Familiar and well-known songs will be sung throughout the sessions such as "Happy together" by The Turtles. Around half way through the sessions, percussion instruments will be given out and used to accompany the around 2-3 songs. The session will often include rounds in which people will be split into smaller groups and each group will sing the same melody in tandem. Lyrics and Lunch is a non-denominational Church-based organisation and a short optional reflective spiritual component is included at the end, but the sessions are open to people with any religion or none. The service is open to PwD living within the community and their carers.

2.2.2. Intervention 2: The Brain Charity's Singing and Dancing Groups

The Brain Charity is based in Liverpool, and they support people with neurological conditions. The charity runs singing (Music Makes Us Sing) and dancing (Music Makes Us Dance) sessions in both community and residential care home settings for PwD and their carers. The singing sessions last approximately one hour and involve engaging in communal singing of familiar songs such as "My Bonnie" by The Beatles lead by two session leaders. Lyrics are presented on a TV screen and attendees are positioned in a semi-circle in view of the screen. The singing sessions have been designed alongside speech and language therapists and focus on speech, language and breathing exercises to improve speech, pitch and rhythm such as call and response and clapping rhythm exercises. Attendees are encouraged to sing and dance along to the songs by session facilitators. The dance sessions last approximately one hour and involve physically engaging chair-based dance moves designed alongside a physical therapist and also incorporates group singing. The sessions are run by two session facilitators who prior to each song will demonstrate simple dance routines to familiar songs such as "Hit the road jack" by Ray Charles. During the song's, session facilitators will give cues to the next dance moves so attendees are able to follow the dance routines. Props will be used throughout the sessions such as scarves, percussion instruments and balloons. The

For peer review only - http://bmjopen.bmj.com/site/about/guidelines.xhtml

sessions are run weekly in 12-week blocks and follow a consistent format each week using similar songs and dance routines so attendees are able to become familiar with them.

2.3. Recruitment and Sample Size

For intervention 1 (Lyrics and Lunch): PwD and carers who have accessed the Lyrics and Lunch groups or who are beginning attending will be invited to participate. A power analysis was conducted using G*Power software version 3.1.9.7. For the analysis, the power level was set at .80 with an error of .05 [62]. The effect size (0.44) was based on a mean effect size taken across eight studies examining active music therapy and reported in Vasionytė et al [63] and based on the primary outcome measure of mood and depressive symptoms. Results revealed a minimum sample size of N= 55 is necessary to achieve a power of .80 at an alpha of .05. Therefore, to account for participant drop out and retention rates, the project will aim to recruit around n=75 PwD and n=75 of their carers from the Lyrics and Lunch groups.

For intervention 2 (The Brain Charity): PwD and carers who have expressed interest in accessing the Brain Charity's singing and dancing groups will be recruited prior to attending the groups. Separate samples will be collected for the singing intervention group and the dancing intervention group. Participants will be recruited from multiple singing and dancing intervention groups across the Liverpool area. These groups will include PwD living within the community and people living in residential care homes. The singing groups and dancing groups will be run by the same session facilitators.

The power analysis remained consistence with intervention 1 and revealed a minimum sample size of N= 55 is necessary to achieve a power of .80 at an alpha of .05. Therefore, across both intervention groups a total sample size of N=300 will be recruited to account for participant drop out. This will consist of n=150 PwD (n=75 from the singing group and n=75 from the dancing group) and n=150 of their carers dementia (n=75 from the singing group and n=75 from the dancing group). Data collection for the study commenced on the 25th May 2023.

2.4. Work Package I – Understanding the impacts of the Lyrics and Lunch support service on people with dementia and their carers

WP1 will address the first research question by assessing the impacts influence of a singing intervention service that includes a sociable lunch aspect on PwD and their carers. This study will use quantitative methods to examine the impact of attending the singing service on cognition, social isolation, loneliness, quality of life, neuropsychiatric symptoms such as

For peer review only - http://bmjopen.bmj.com/site/about/guidelines.xhtml

agitation or other responsive behaviours, mood (depression/anxiety) and carer burden. Additionally, the core outcome set of 13 outcomes of interventions valued by people living at home with dementia, developed directly with PwD, informal and professional carers [64] will be used. The 13 outcomes were classified into 4 domains including friendly neighbourhood and home, independence, self-managing dementia symptoms and quality of life and will be examined using a bespoke quantitative measure, an 18-item measure adapted from the Adults Social Care Outcome assessment [65], the engagement and independence in dementia questionnaire [66] and Older Americans' Resources and Services Instrumental Activities of Daily Living assessment [67]. Outcome measures will be examined when the sessions are running (ON session) and then compare this to periods of time when the session is not running such as summer holidays and Christmas breaks (OFF session) (Table 1). Participants will be required to have attended the Lyrics and Lunch sessions for a minimum period of 4 weeks prior to the ON session assessment. OFF session assessments will be completed after the sessions have not been attended for a minimum of 4 weeks. The Lyrics and Lunch sessions run weekly or biweekly.

2.4.1. Measures

Standard outcome measures that will be assessed in PwD and their carers will include: cognition assessed using a shortened version of the Addenbrooke's cognitive examination (ACE-III) [68] were participant's verbal fluency and attention will be assessed, quality of life assessed using the dementia quality of life measure (DemQOL and DemQOL-Proxy) [69], social Isolation assessed using the Duke Social Support Index (DSSI-10) [70], loneliness assessed using Three-item loneliness UCLA Measure [71], mood and depressive symptoms assessed using the Quick Inventory of Depressive Symptomatology (QIDS-SR) [72], neuropsychiatric symptoms assessed using Neuropsychiatric Inventory Questionnaire (NPI-Q) completed by carers [73] and carer burden assessed using the Zarit burden questionnaire[74].

Demographic information will be collected from participants at the first assessment including age, gender, ethnicity, highest education level (an indicator of SEP), number of years in formal education (an indicator of SEP), years since diagnosis and years since symptom onset. Participant attendance will be recorded to determine adherence to the intervention. The Global Deterioration Scale for Assessment of Primary Degenerative Dementia will be completed by carers to determine dementia severity [75].

For peer review only - http://bmjopen.bmj.com/site/about/guidelines.xhtml

It is hypothesised that attendance to singing interventions will increase quality of life and mood and reduce depressive and neuropsychiatric and symptoms in PwD and their carers. Social isolation can be prominent in PwD and attending the singing intervention may reduce feelings of social isolation. Due to this, it is hypothesised that attending the singing intervention will result in reduced self-reported feelings of social isolation and loneliness in both PwD and their carers. It is hypothesised that both PwD and their carers will show improvements on the core outcome measures during the times that they are attending the singing intervention compared to period when they are not attending the sessions.

2.4.2. Data Analysis

To examine whether there were changes in outcome variables during on and off periods and to assess whether singing intervention support services are associated with changes in mood/depressive symptoms, quality of life, cognition and social isolation in people living with dementia and their carers, linear regression models will be used with time (on vs off) as the exposure variable. For the model examining outcomes in people living with dementia, outcome variables will be QIDS-SR score, ACE-III score, DemQOL score, DSSI-10 score, NPI-Q score and Core outcome set score. Covariates will include participant age, years since diagnosis, years since symptom onset and number of sessions attended. A separate model will examine outcome measures in dementia carers, outcome measures will be DSSI-10 score, QIDS-SR score and carer burden.

2.5. Work Package II – Understanding the impacts of singing and dancing support service conducted by The Brain Charity on people with dementia and their carers

WP2 will address the second research question and the impacts of attending either a 12-week singing intervention or a 12-week dancing intervention that also involves some singing for PwD and their carers. Participants will only attend one intervention service (either the singing or the dancing intervention). The benefits of dementia singing and dancing groups for both PwD and their unpaid carers, both community-residing and those living in care homes (conducted within the care home), will be examined using a mixed-methods approach. The project will assess the influence of the services on cognition, mood, social isolation, loneliness, carer burden and neuropsychiatric symptoms.

2.5.1. Measures

For peer review only - http://bmjopen.bmj.com/site/about/guidelines.xhtml

The following outcome measures will be used: DemQol, DemQol-Proxy, QIDS-SR, ACE-III (shortened version assessing verbal fluency and attention only), NPI-Q, DSSI-10, Zarit burden questionnaire (Figure 1). Outcome measures will be examined at baseline (before the start of the 12-week block of sessions) and then one week after the final session, thus providing pre and post-intervention measures. The QIDS-SR will be completed every four weeks during the 12 weeks to examine more subtle changes in mood and depressive symptoms over the 12 weeks (Figure 1). The dance elements included in the dancing sessions may have impacts on the physical health and frailty of PwD attending the sessions. Due to this, The Physical Frailty Phenotype [76] measure of physical frailty will be included for the dancing group that will assess weight loss, grip strength, exhaustion, gait speed and physical activity. The Physical Frailty Phenotype will be completed pre and post intervention.

Similar to WP1, participant attendance will be recorded to determine adherence to the intervention. Demographic information will be collected at baseline, including age, gender, ethnicity, highest education level (an indicator of SEP), number of years in formal education (an indicator of SEP), years since diagnosis and years since symptom onset. The Global Deterioration Scale for Assessment of Primary Degenerative Dementia will be completed by carers to determine dementia severity [75].

2.5.2 Data Analysis

Consistent with WP1, we will examine whether there were changes in outcome variables pre vs. post-intervention and assess whether singing and dancing interventions are associated with changes in mood/depressive symptoms, quality of life, cognition and social isolation in people living with dementia and their carers. Linear regression models will be used with time (pre/post) and intervention type (singing or dancing intervention) as the exposure variables. The outcome variables will be QIDS-SR score, ACE-III score, DemQOL score, DSSI-10 score, Zarit burden score, NPI-Q score, sit-to-stand assessment score and gait assessment score. Covariates will include participant age, years since diagnosis, years since symptom onset, ACE score (dementia severity) and number of sessions attended. Separate models will be conducted to examine the singing and dancing services effect on people living with dementia and then their carers and for each of the outcome measures.

2.6. Work Package III – Understanding the acceptability, remote delivery and barriers to accessing and continued engagement with singing interventions

For peer review only - http://bmjopen.bmj.com/site/about/guidelines.xhtml

WP3 will address research questions 3 and 4 and will examine barriers to accessing and continued engagement with singing interventions and how these factors may differ based on SEP and location (urban vs rural location). Accessibility barriers are likely to vary across these areas and depend on people's SEP and should be examined and acknowledged when rolling out intervention programmes on a wider scale.

A series of semi-structured follow-up interviews will be conducted with a subset of PwD, unpaid carers, service providers (music therapists, workshop coordinators) and local facilitators (paid carers, care home managers). The topic guide(s) will be coproduced with the teams and four public advisers consisting of a person living with dementia, a paid carer, an unpaid carer and a music teacher (JM) who runs the sessions.

The first aim will focus on barriers to access and continued engagement with the intervention service. To address this, a subset of 20 people will be recruited (approx. 10 PwD and 10 unpaid carers) from both interventions, resulting in a total of 40 interviews. This initial sample size determination was guided by information power methods [77]. These interviews will focus on the following topics: acceptability of the services, impacts of the service on mood and overall quality of life and accessibility including barriers to access and continued engagement.

Our second aim is to examine the potential of services to be adapted for remote delivery and potential barriers. When discussing remote access, adaptations made to the services when COVID-19 restrictions were in place will be discusses and how successful these remote access adaptations were. To address this, 20 people will be recruited, approx. 10 service providers (music therapists, workshop coordinators) and 10 local facilitators (paid carers, care home managers). These interviews will focus on the following topics: impacts of the service on PwD and their carers, and barriers to remote delivery of singing. Again, this initial sample size determination was guided by information power methods [77]. Preliminary analysis will be used to assess power of the analysis during the data collection phase to determine if the sample size calculation is correct. After the first three interviews, a first review of the data will be conducted, and first suggestions of relevant theory/themes will be made. This assessment will be considered again before closing data collection. Data will be analysed using Braun & Clarke's reflexive thematic analysis [78], and public advisors will assist with coding the transcripts.

For peer review only - http://bmjopen.bmj.com/site/about/guidelines.xhtml

2.7. Work Package IV – Examination of the social impact of singing interventions

WP4 will address research question 5 and the social impact of singing interventions examined by conducting an evaluative social return on investment (SROI) analysis on both services. Using standardised methods and following the seven principles a SROI will be conducted [79]. Separate SROI analyses will be conducted for the Lyrics and Lunch Charity and The Brain Charity. The methods and procedure employed for both analyses will remain consistent. An impact map will be created to calculate the social value generated by the intervention service which involves six stages [80]. Stage 1 will identify key stakeholders and people likely to be impacted by the services such as PwD, relatives, unpaid and paid carers, Lyrics and Lunch and The Brain Charity. Stage 2 will use quantitative data collected for WPs I and II on the effects of the intervention on quality of life, mood, carer burden, depressive symptoms and agitation levels and will map identified outcomes creating an impact map. This stage will identify what changes occurred and for whom. Stage 3 will provide a monetised value to each of the outcomes including those that do not have a price attached to them such as changes in quality of life. For outcome measures that do not have a clear monitory value, established SROI value banks such as Social Value UK will be used to identify suitable financial proxies [81]. Stage 4 will establish impact by accounting for attribution, deadweight, displacement and drop-off. These adjustments are made to endure that social value is not overclaimed [79]. Percentage values will be determined for attribution which considers changes or outcomes declared in the analysis that may not be due to just the singing intervention but may be due to other support services. A percentage value for the deadweight will be determined which considers how much change in the outcome values would have happened regardless of the singing intervention programme. Deadweight will be calculated as a 10% reduction in overall value for every resource the participant identifies as an alternative to the singing support service, for example if participants attend other support groups. Stage 5 will calculate the SROI benefits by adding up and subtracting any negatives (attribution, distribution, drop-off and deadweight), and this will be compared to the investment cost of the service. This calculation will provide us with a SROI ratio which demonstrates the social value of the service in relation to the cost invested, for example an SROI ratio of £3.50: £1 means that for every pound invested there is £3.50 of social value created. Stage 6 will disseminate the findings with key stakeholders and recommendations created for future delivery of the service.

2.8. Patient and Public Involvement

For peer review only - http://bmjopen.bmj.com/site/about/guidelines.xhtml

This project has been developed and conceptualised alongside members of the public and key stakeholders. Our formal project team includes four public advisors including a person living with dementia (SM), two carers (one paid (SP) and one unpaid (HB)) and also a music teacher (JM) who runs the singing sessions. All public advisors have previous experience and involvement with the singing sessions provided by The Brain Charity or the Lyrics and Lunch Charity. Public advisors contribute to all aspects of this research project, from designing study documents and advising on outcome measures to the interpretation of findings and dissemination of the research. Public advisors are reimbursed for the time they contribute to the project.

2.9. Ethics and Dissemination

This project has received ethical approval from the University of Liverpool's Ethics Committee (App ref: 12374) and Lancaster University's Ethics Committee (App ref: 3442). All methods will be conducted in accordance with the relevant guidelines and regulations and all participants will provide informed consent to participate. Findings from this project will be presented at national and international conferences and published in scientific journals. Findings will be publicly disseminated to key stakeholders and within the community and the organisations included in this study will use the outcomes of the research for their own continuous improvement strategies.

3.0. Discussion

Using a mixed-methods design, this project seeks to explore the influence of two established singing intervention services for PwD and their carers. The study aims to collect a large sample of key stakeholders to assess the influence of attending singing support services on well-being and quality of life through a combination of quantitative and qualitative methods. The findings from this research will highlight the impact of singing support services for PwD and their carers and will allow the SROI of such services to be evaluated. The project will examine the barriers that people encounter when accessing and continuing to engage with singing interventions and whether these barriers disproportionately affect people from lower SEP. It can be challenging to access support services after a diagnosis of dementia [56] and exploring ways to make services more widely and easily accessible is important. Methods to reduce potential barriers to access such as travel, increase reliance on carers and availability of services will be explored alongside the potential for remote delivery of singing services to improve and expand access.

For peer review only

A limitation of this study that should be noted is the lack of an independent control group. This study is therefore unable to robustly examine the impact of each intervention. However, it will provide important information on the acceptability and potential impact of the support service for PwD and their careers and barriers to access or engagement. This study will provide an important knowledge base for future studies and provide evidence whether larger randomised controls studies are warranted. A further limitation is that as participants cannot be blinded, self-report measures may include an element of bias in the responses.

If the results from the current study suggest that singing support services have a positive impact on PwD and their carers, then support services could be scaled up and applied to other regions and settings to improve access to beneficial support services. Examining the SROI will provide an insight into the cost effectiveness of the support services and if they will be suitable to apply in lower income areas and regions both nationally and internationally. The findings from this project will be used to develop recommendations for future delivery of singing intervention services.

Abbreviation List

PwD	People living with dementia
WP	Work package
SEP	Socioeconomic Position
DemQOL	Dementia quality of life measure
ACE-III	Addenbrooke's cognitive examination
DSSI-10	Duke Social Support Index
QIDS-SR	Quick Inventory of Depressive Symptomatology
NPI-Q	Neuropsychiatric Inventory Questionnaire
SROI	Social return on investment

Conflicts of Interest: Carol Holland, Jeanette Main and Steve Pendrill are trustees of the Lyrics and Lunch Charity. All other authors declare no conflict of interest.

Consent for publication: N/A

Funding: National Institute for Health and Care Research Applied Research Collaboration North West Coast (ARC NWC) (Award/Grant no: NA) and The Alzheimer's Society (Award/Grant no: NA).

For peer review only - http://bmjopen.bmj.com/site/about/guidelines.xhtml

Author Contributions: Conceptualisation and methodology design contributions and project administration contributions were made by all authors (MP, KH, KW, FA, HB, CH, HB, JM, SM, SP, CG). The original draft preparation contributions were made by MP. MP, KH, KW, FA, HB, CH contributed to revising the manuscript. All authors (MP, KH, KW, RA, HB, CH, HB, JM, SM, SP, CG) read and agreed to the published version of the manuscript.

Availability of data and materials: Data generated in this study will be made available on Open Science Framework upon completion of the study.

Acknowledgements: N/A

For peer review only

References

1. World Health Organization . Geneva, Switzerland: World Health Organization; 2021. Fact sheets of dementia [Internet]. Available from: https:// www.who.int/news-room/fact-sheets/detail/dementia
2. Sliwinski M, Lipton RB, Buschke H, Stewart W. The effects of preclinical dementia on estimates of normal cognitive functioning in aging. The Journals of Gerontology Series B: Psychological Sciences and Social Sciences. 1996 Jul 1;51(4):P217-25.
3. Srikanth S, Nagaraja AV, Ratnavalli E. Neuropsychiatric symptoms in dementia-frequency, relationship to dementia severity and comparison in Alzheimer's disease, vascular dementia and frontotemporal dementia. Journal of the neurological sciences. 2005 Sep 15;236(1-2):43-8.
4. Cagnin A, Gnoato F, Jelcic N, Favaretto S, Zarantonello G, Ermani M, Dam M. Clinical and cognitive correlates of visual hallucinations in dementia with Lewy bodies. Journal of Neurology, Neurosurgery & Psychiatry. 2013 May 1;84(5):505-10.
5. Kwak YT, Yang Y, Koo MS. Anxiety in dementia. Dementia and neurocognitive disorders. 2017 Jun;16(2):33.
6. Banerjee S, Smith SC, Lamping DL, Harwood RH, Foley B, Smith P, Murray J, Prince M, Levin E, Mann A, Knapp M. Quality of life in dementia: more than just cognition. An analysis of associations with quality of life in dementia. Journal of Neurology, Neurosurgery & Psychiatry. 2006 Feb 1;77(2):146-8.
7. Giebel CM, Sutcliffe C, Challis D. Hierarchical decline of the initiative and performance of complex activities of daily living in dementia. Journal of geriatric psychiatry and neurology. 2017;30(2):96-103.
8. Resnick B, Galik E, Kolanowski A, VanHaitsma K, Boltz M, Zhu S, Ellis J, Behrens L, Eshraghi K, Renn C, Dorsey SG. The relationship between pain, function, behavioral, and psychological symptoms of dementia and quality of life. Pain Management Nursing. 2022 Feb 1;23(1):55-61.
9. Watts KE, Storr NJ, Barr PG, Rajkumar AP. Systematic review of pharmacological interventions for people with Lewy body dementia. Aging & Mental Health. 2023 Feb 1;27(2):203-16.
10. Gerlach LB, Kales HC. Pharmacological management of neuropsychiatric symptoms of dementia. Current treatment options in psychiatry. 2020 Dec;7:489-507.

For peer review only - http://bmjopen.bmj.com/site/about/guidelines.xhtml

11. Gustafsson M, Karlsson S, Gustafson Y, Lövheim H. Psychotropic drug use among people with dementia–a six-month follow-up study. BMC Pharmacology and Toxicology. 2013 Dec;14(1):1-9.
12. Maidment ID, Damery S, Campbell N, Seare N, Fox C, Iliffe S, Hilton A, Brown G, Barnes N, Wilcock J, Randle E. Medication review plus person-centred care: a feasibility study of a pharmacy-health psychology dual intervention to improve care for people living with dementia. BMC psychiatry. 2018 Dec;18(1):1-1.
13. Maust DT, Kim HM, Seyfried LS, Chiang C, Kavanagh J, Schneider LS, Kales HC. Antipsychotics, other psychotropics, and the risk of death in patients with dementia: number needed to harm. JAMA psychiatry. 2015 May 1;72(5):438-45.
14. Tampi RR, Tampi DJ, Balachandran S, Srinivasan S. Antipsychotic use in dementia: a systematic review of benefits and risks from meta-analyses. Therapeutic advances in chronic disease. 2016 Sep;7(5):229-45.
15. Ballard C, Corbett A. Management of neuropsychiatric symptoms in people with dementia. CNS drugs. 2010 Sep;24:729-39.
16. Maidment ID, Shaw RL, Killick K, Damery S, Hilton A, Wilcock J, Barnes N, Brown G, Gillespie S, Fox C, Barton G. Improving the management of behaviour that challenges associated with dementia in care homes: protocol for pharmacy–health psychology intervention feasibility study. BMJ open. 2016 Mar 1;6(3):e010279.
17. Rochon PA, Normand SL, Gomes T, Gill SS, Anderson GM, Melo M, Sykora K, Lipscombe L, Bell CM, Gurwitz JH. Antipsychotic therapy and short-term serious events in older adults with dementia. Archives of internal medicine. 2008 May 26;168(10):1090-6.
18. O'Donnell E, Holland C, Swarbrick C. Strategies used by care home staff to manage behaviour that challenges in dementia: A systematic review of qualitative studies. International Journal of Nursing Studies. 2022 Apr 22:104260.
19. Moreno-Morales C, Calero R, Moreno-Morales P, Pintado C. Music therapy in the treatment of dementia: A systematic review and meta-analysis. Frontiers in medicine. 2020 May 19;7:160.
20. Dimitriou TD, Verykouki E, Papatriantafyllou J, Konsta A, Kazis D, Tsolaki M. Non-Pharmacological interventions for the anxiety in patients with dementia. A cross-over randomised controlled trial. Behavioural brain research. 2020 Jul 15;390:112617.
21. Clair AA, Memmott J. Therapeutic uses of music with older adults. American Music Therapy Association. 8455 Colesville Road Suite 1000, Silver Spring, MD 20910; 2008.
22. Davidson JW, Almeida RA. An exploratory study of the impact of group singing activities on lucidity, energy, focus, mood and relaxation for persons with dementia and their caregivers. Psychology of Well-Being. 2014 Dec;4(1):1-3.
23. Lee S, O'Neill D, Moss H. Promoting well-being among people with early-stage dementia and their family carers through community-based group singing: a phenomenological study. Arts & Health. 2022a Jan 2;14(1):85-101.
24. Lee S, Allison T, O'Neill D, Punch P, Helitzer E, Moss H. Integrative review of singing and music interventions for family carers of people living with dementia. Health Promotion International. 2022b May;37(Supplement_1):i49-61.
25. Jeffries, K. J., Fritz, J. B., & Braun, A. R. (2003). Words in melody: an H215O PET study of brain activation during singing and speaking. *Neuroreport*, *14*(5), 749-754.
26. Carruth EK. The effects of singing and the spaced retrieval technique on improving face-name recognition in nursing home residents with memory loss. Journal of Music Therapy. 1997 Oct 1;34(3):165-86.
27. Satoh M, Yuba T, Tabei KI, Okubo Y, Kida H, Sakuma H, Tomimoto H. Music therapy using singing training improves psychomotor speed in patients with Alzheimer's disease: a neuropsychological and fMRI study. Dementia and geriatric cognitive disorders extra. 2015;5(3):296-308.
28. Mansens D, Deeg DJ, Comijs HC. The association between singing and/or playing a musical instrument and cognitive functions in older adults. Aging & Mental Health. 2018 Aug 3;22(8):970-7.

For peer review only - http://bmjopen.bmj.com/site/about/guidelines.xhtml

29. Lin Y, Chu H, Yang CY, Chen CH, Chen SG, Chang HJ, Hsieh CJ, Chou KR. Effectiveness of group music intervention against agitated behavior in elderly persons with dementia. International journal of geriatric psychiatry. 2011 Jul;26(7):670-8.
30. Unadkat S, Camic PM, Vella-Burrows T. Understanding the experience of group singing for couples where one partner has a diagnosis of dementia. The Gerontologist. 2017 Jun 1;57(3):469-78.
31. Li HC, Wang HH, Chou FH, Chen KM. The effect of music therapy on cognitive functioning among older adults: A systematic review and meta-analysis. Journal of the American Medical Directors Association. 2015 Jan 1;16(1):71-7.
32. Lam HL, Li WT, Laher I, Wong RY. Effects of music therapy on patients with dementia—A systematic review. Geriatrics. 2020 Sep 25;5(4):62.
33. Dowson B, Schneider J. Online singing groups for people with dementia: scoping review. Public Health. 2021 May 1;194:196-201.
34. McDermott O, Crellin N, Ridder HM, Orrell M. Music therapy in dementia: a narrative synthesis systematic review. International journal of geriatric psychiatry. 2013 Aug;28(8):781-94.
35. Cho HK. The effects of music therapy-singing group on quality of life and affect of persons with dementia: a randomized controlled trial. Frontiers in medicine. 2018 Oct 15;5:279.
36. Baker FA, Lee YE, Sousa TV, Stretton-Smith PA, Tamplin J, Sveinsdottir V, Geretsegger M, Wake JD, Assmus J, Gold C. Clinical effectiveness of music interventions for dementia and depression in elderly care (MIDDEL): Australian cohort of an international pragmatic cluster-randomised controlled trial. The Lancet Healthy Longevity. 2022 Mar 1;3(3):e153-65.
37. Blankevoort CG, Van Heuvelen MJ, Boersma F, Luning H, De Jong J, Scherder EJ. Review of effects of physical activity on strength, balance, mobility and ADL performance in elderly subjects with dementia. Dementia and geriatric cognitive disorders. 2010 Oct 28;30(5):392-402.
38. Brasure M, Desai P, Davila H, Nelson VA, Calvert C, Jutkowitz E, Butler M, Fink HA, Ratner E, Hemmy LS, McCarten JR. Physical activity interventions in preventing cognitive decline and Alzheimer-type dementia: a systematic review. Annals of internal medicine. 2018 Jan 2;168(1):30-8.
39. Kontos P, Grigorovich A, Kosurko A, Bar RJ, Herron RV, Menec VH, Skinner MW. Dancing with dementia: Exploring the embodied dimensions of creativity and social engagement. The Gerontologist. 2021 Aug;61(5):714-23.
40. Salihu D, Wong EM, Bello UM, Kwan RY. Effects of dance intervention on agitation and cognitive functioning of people living with dementia in institutional care facilities: Systematic review. Geriatric Nursing. 2021 Nov 1;42(6):1332-40.
41. Bracco L, Poirier G, Pinto-Carral A, Mourey F. Effect of Dance Therapy on the Physical Abilities of Older Adults with Dementia: A Systematic Review. Eur J Geriatr Gerontol. 2021 Dec 1;3(3):134-42.
42. Fratiglioni L, Wang HX. Brain reserve hypothesis in dementia. Journal of Alzheimer's disease. 2007 Jan 1;12(1):11-22.
43. Adkins-Regan E. Activity dependent brain plasticity: does singing increase the volume of a song system nucleus? Theoretical comment on Sartor and Ball (2005).
44. Galinha I, Pinal D, Lima ML, Labisa-Palmeira A. The role of social and physiological variables on older adults' cognitive improvement after a group singing intervention: the Sing4health randomized controlled trial. Psychosocial Intervention. 2021;30(3):123-38.
45. Clark IN, Tamplin JD, Baker FA. Community-dwelling people living with dementia and their family caregivers experience enhanced relationships and feelings of well-being following therapeutic group singing: A qualitative thematic analysis. Frontiers in Psychology. 2018 Jul 30;9:1332.
46. Dickinson C, Dow J, Gibson G, Hayes L, Robalino S, Robinson L. Psychosocial intervention for carers of people with dementia: what components are most effective and when? A systematic review of systematic reviews. International psychogeriatrics. 2017 Jan;29(1):31-43.

For peer review only - http://bmjopen.bmj.com/site/about/guidelines.xhtml

47. Farina N, Page TE, Daley S, Brown A, Bowling A, Basset T, Livingston G, Knapp M, Murray J, Banerjee S. Factors associated with the quality of life of family carers of people with dementia: A systematic review. Alzheimer's & Dementia. 2017 May 1;13(5):572-81.
48. Laver K, Milte R, Dyer S, Crotty M. A systematic review and meta-analysis comparing carer focused and dyadic multicomponent interventions for carers of people with dementia. Journal of Aging and Health. 2017 Dec;29(8):1308-49.
49. Teahan A, Lafferty A, McAuliffe E, Phelan A, O'Sullivan L, O'Shea D, Nicholson E, Fealy G. Psychosocial interventions for family carers of people with dementia: A systematic review and meta-analysis. Journal of Aging and Health. 2020 Oct;32(9):1198-213.
50. Lee S, Allison T, O'Neill D, Punch P, Helitzer E, Moss H. Integrative review of singing and music interventions for family carers of people living with dementia. Health Promotion International. 2022 May;37(Supplement_1):i49-61.
51. Sutcliffe C, Giebel C, Bleijlevens M, Lethin C, Stolt M, Saks K, Soto ME, Meyer G, Zabalegui A, Chester H, Challis D. Caring for a person with dementia on the margins of long-term care: a perspective on burden from 8 European countries. Journal of the American Medical Directors Association. 2017 Nov 1;18(11):967-73.
52. Alwin J, Öberg B, Krevers B. Support/services among family caregivers of persons with dementia—perceived importance and services received. International Journal of Geriatric Psychiatry: A journal of the psychiatry of late life and allied sciences. 2010 Mar;25(3):240-8.
53. Bayly M, Morgan D, Chow AF, Kosteniuk J, Elliot V. Dementia-related education and support service availability, accessibility, and use in rural areas: Barriers and solutions. Canadian Journal on Aging/La Revue canadienne du vieillissement. 2020 Dec;39(4):545-85.
54. Morgan DG, Semchuk KM, Stewart NJ, D'arcy C. Rural families caring for a relative with dementia: Barriers to use of formal services. Social science & medicine. 2002 Oct 1;55(7):1129-42.
55. Hossain MZ, Khan HT. Barriers to access and ways to improve dementia services for a minority ethnic group in England. Journal of evaluation in clinical practice. 2020 Dec;26(6):1629-37.
56. Giebel C, Robertson S, Beaulen A, Zwakhalen S, Allen D, Verbeek H. "Nobody Seems to Know Where to Even Turn To": Barriers in Accessing and Utilising Dementia Care Services in England and The Netherlands. International Journal of Environmental Research and Public Health. 2021 Nov 22;18(22):12233.
57. Dowson B, Schneider J. Online singing groups for people with dementia: scoping review. Public Health. 2021 May 1;194:196-201.
58. Lee S, O'Neill D, Moss H. Dementia-inclusive group-singing online during COVID-19: A qualitative exploration. Nordic Journal of Music Therapy. 2022 Aug 8;31(4):308-26.
59. Hopkins G, Winrow E, Davies C, Seddon D. Beyond social prescribing—The use of social return on investment (SROI) analysis in integrated health and social care interventions in England and Wales: A protocol for a systematic review. Plos one. 2023 Feb 21;18(2):e0277386.
60. Jones C, Windle G, Edwards RT. Dementia and imagination: a social return on investment analysis framework for art activities for people living with dementia. The Gerontologist. 2020 Jan 24;60(1):112-23.
61. Bosco A, Schneider J, Broome E. The social value of the arts for care home residents in England: a social return on investment (SROI) analysis of the imagine arts programme. Maturitas. 2019 Jun 1;124:15-24.
62. Faul F, Erdfelder E, Lang AG, Buchner A. G* Power 3: A flexible statistical power analysis program for the social, behavioral, and biomedical sciences. Behavior research methods. 2007 May;39(2):175-91.
63. Vasionytė I, Madison G. Musical intervention for patients with dementia: a meta-analysis. Journal of clinical nursing. 2013 May;22(9-10):1203-16.
64. Reilly ST, Harding AJ, Morbey H, Ahmed F, Williamson PR, Swarbrick C, Leroi I, Davies L, Reeves D, Holland F, Hann M. What is important to people with dementia living at home? A

For peer review only - http://bmjopen.bmj.com/site/about/guidelines.xhtml

set of core outcome items for use in the evaluation of non-pharmacological community-based health and social care interventions. Age and ageing. 2020 Jul 1;49(4):664-71.
65. Johnstone L, Page C. Using Adult Social Care Outcomes Toolkit (ASCOT) in the assessment and review process. Res Policy Plann. 2013;30(3):179-92.
66. Stoner, C.R., Orrell, M., & Spector, A. (2018). Psychometric properties and factor analysis of the Engagement and Independence in Dementia Questionnaire (EID-Q). Dementia and Geriatric Cognitive Disorders, 46, 3-4, 119 – 127. DOI:10.1159/000488484
67. Doble SE, Fisher AG. The dimensionality and validity of the Older Americans Resources and Services (OARS) Activities of Daily Living (ADL) scale. Journal of Outcome Measurement. 1998 Jan 1;2(1):4-24.
68. Hsieh S, Schubert S, Hoon C, Mioshi E, Hodges JR. Validation of the Addenbrooke's Cognitive Examination III in frontotemporal dementia and Alzheimer's disease. Dementia and geriatric cognitive disorders. 2013;36(3-4):242-50.
69. Smith SC, Lamping DL, Banerjee S, Harwood R, Foley B, Smith P, Cook JC, Murray J, Prince M, Levin E, Mann A. Measurement of health-related quality of life for people with dementia: development of a new instrument (DEMQOL) and an evaluation of current methodology. Health Technology Assessment (Winchester, England). 2005;9(10):1-iv.
70. Koenig HG, Westlund RE, George LK, Hughes DC, Blazer DG, Hybels C. Abbreviating the Duke Social Support Index for use in chronically ill elderly individuals. Psychosomatics. 1993 Jan 1;34(1):61-9.
71. Russell DW. UCLA Loneliness Scale (Version 3): Reliability, validity, and factor structure. Journal of personality assessment. 1996 Feb 1;66(1):20-40.
72. Rush AJ, Trivedi MH, Ibrahim HM, Carmody TJ, Arnow B, Klein DN, Markowitz JC, Ninan PT, Kornstein S, Manber R, Thase ME. The 16-Item Quick Inventory of Depressive Symptomatology (QIDS), clinician rating (QIDS-C), and self-report (QIDS-SR): a psychometric evaluation in patients with chronic major depression. Biological psychiatry. 2003 Sep 1;54(5):573-83.
73. Kaufer DI, Cummings JL, Ketchel P, Smith V, MacMillan A, Shelley T, Lopez OL, DeKosky ST. Validation of the NPI-Q, a brief clinical form of the Neuropsychiatric Inventory. The Journal of neuropsychiatry and clinical neurosciences. 2000 May;12(2):233-9.
74. Yap P. Validity and reliability of the Zarit Burden Interview in assessing caregiving burden. Ann Acad Med Singapore. 2010;39:758-63.0 May;12(2):233-9.
75. Reisberg B, Ferris SH, de Leon MJ, Crook T. Global Deterioration Scale. The American Journal of Psychiatry. 1983.
76. Fried LP, Tangen CM, Walston JD, Newman AB, Hirsch C, Gottdiener JS. E Seeman, T.; Tracy, RP; Kop, WJ; Burke, GL; et al. Frailty in older adults: Evidence for a phenotype. J. Gerontol. Ser. A Boil. Sci. Med. Sci. 2001;56:M146-57.
77. Malterud K, Siersma VD, Guassora AD. Sample size in qualitative interview studies: guided by information power. Qualitative health research. 2016 Nov;26(13):1753-60.
78. Braun, V., & Clarke, V. (2021). One size fits all? What counts as quality practice in (reflexive) thematic analysis?. *Qualitative research in psychology*, *18*(3), 328-352.
79. Fujiwara D. The seven principle problems of SROI. London: Simetrica Ltd. 2015 Aug.
80. Banke-Thomas AO, Madaj B, Charles A, van den Broek N. Social Return on Investment (SROI) methodology to account for value for money of public health interventions: a systematic review. BMC public health. 2015 Dec;15(1):1-4.
81. Social Value UK/HACT. SROI and HACT Social Value Bank Linkages Paper—Social Value UK. Social Value UK. 2015. [accessed 7 Oct 2022] Available from: https://socialvalueuk.org/resource/sroi-and-hact-social-value-bank-linkages-paper/

For peer review only - http://bmjopen.bmj.com/site/about/guidelines.xhtml

Tables

Table 1. Assessments to be completed by PwD and their carers

Assessment Time period	Assessment completed by Person living with Dementia	Assessment completed by unpaid carer
**ON session (Participant has attended service for a minimum of 4 weeks)**	QIDS-SR, ACE, DemQOL, DSSI-10	QIDS-SR, DSSI-10, NPI-Q, DemQOL-Proxy, Zarit burden questionnaire
**OFF session (Participant has not attended a session for a minimum of 4 weeks)**	QIDS-SR, ACE, DemQOL, DSSI-10	QIDS-SR, DSSI-10, NPI-Q, DemQOL-Proxy, Zarit burden questionnaire

For peer review only

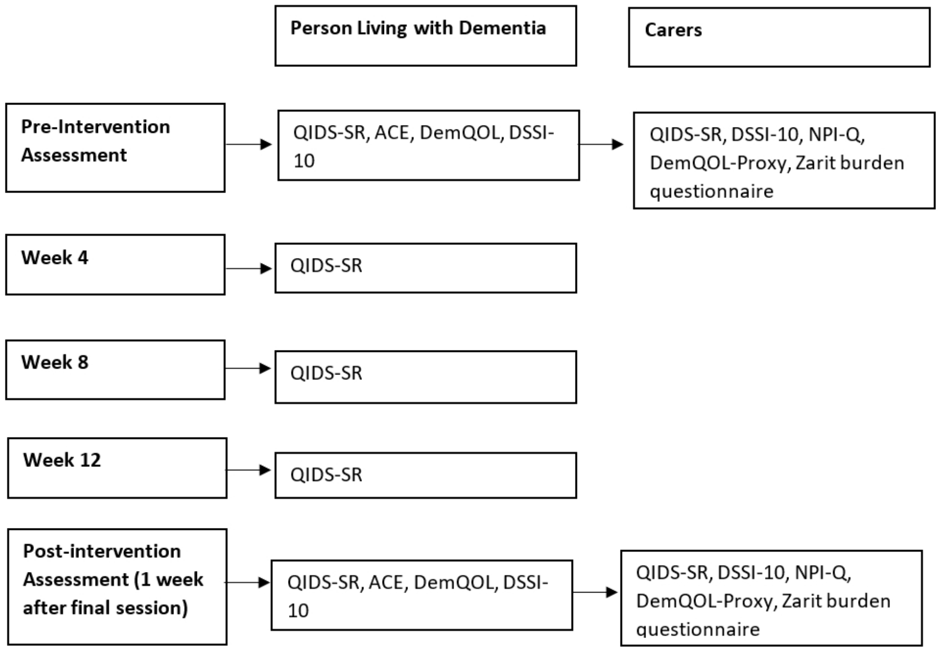

Figure 1. Timeline of cognitive assessments for PwD and unpaid carers

682x470mm (38 x 38 DPI)

BMJ Open

Do community-based singing interventions have an impact on people living with dementia and their carers? A mixed-methods study protocol

Journal:	*BMJ Open*
Manuscript ID	bmjopen-2023-076168.R2
Article Type:	Protocol
Date Submitted by the Author:	23-Oct-2023
Complete List of Authors:	Polden, Megan; University of Liverpool, Department of Primary Care & Mental Health; Lancaster University<br>Hanna, Kerry; University of Liverpool, School of health sciences<br>Ward, Kym; The Brain Charity<br>Ahmed, Faraz; Lancaster University<br>Brown, Heather; Lancaster University, Division of Health Research<br>Holland, Carol ; Lancaster University, Ageing<br>Barrow, Hazel; NIHR Applied Research Collaboration North West Coast<br>Main, Jeanette; Lyrics and Lunch Charity<br>Mann, Stella; NIHR Applied Research Collaboration North West Coast<br>Pendrill, Steve; Lyrics and Lunch Charity<br>Giebel, Clarissa; University of Liverpool, Institute of Psychology, Health and Society
**Primary Subject Heading**:	Sociology
Secondary Subject Heading:	Mental health, Sociology, Public health
Keywords:	Dementia < NEUROLOGY, Aging, MENTAL HEALTH

SCHOLARONE™
Manuscripts

Do community-based singing interventions have an impact on people living with dementia and their carers? A mixed-methods study protocol

Megan Polden[1,2,5*], Kerry Hanna[3], Kym Ward[2,4], Faraz Ahmed[5], Heather Brown[5], Carol Holland[5,6], Hazel Barrow[2], Jeanette Main[2,6], Stella Mann[2], Steve Pendrill[2,6], Clarissa Giebel[1,2]

1 Department of Primary Care & Mental Health, University of Liverpool, Liverpool, UK

2 NIHR Applied Research Collaboration North West Coast, Liverpool, UK

3 School of Health Sciences, University of Liverpool, UK

4 The Brain Charity, Liverpool, UK

5 Health Research, Lancaster University, UK

6 Lyrics and Lunch Charity, UK

*Correspondence to:

Megan Polden

Email: m.polden@liverpool.ac.uk

For peer review only

Word count: 5088

Abstract

Introduction: Psychosocial interventions have been shown to improve mood, relieve stress and improve quality of life for people living with dementia (PwD). To date, most evaluations of singing interventions have focused on the benefits for PwD and not their carers. This research aims to evaluate the benefits of dementia singing groups for both PwD and their carers.

Methods and analysis: This 2-year project will observe the impact of two different singing intervention services, one combining singing alongside dance and another that includes a

sociable lunch. This project will aim to recruit a total of n=150 PwD and n=150 carers across the two singing interventions. Using a mixed-methods approach, the influence of both services will be analysed via the following outcome measures: quality of life, neuropsychiatric symptoms, social isolation, loneliness, cognition, carer burden and depressive symptoms in PwD and their carers using a pre/post study design. Regression models will be used to analyse the data with time (pre/post) as the exposure variable. Semi-structured interviews will be conducted with a subset of people (n=40) to further investigate the impact of singing services with a specific focus on the acceptability of the interventions, barriers to access and prolonged engagement and potential for remote delivery. Interview data will be analysed using Braun & Clarke's reflexive thematic analysis, and public advisors will assist with coding the transcripts. A social return on investment analysis will be conducted to determine the social impact of the services.

Ethics and dissemination: This project has received ethical approval from the University of Liverpool's Ethics Committee (App ref: 12374) and Lancaster University's Ethics Committee (App ref: 3442). All participants will provide informed consent to participate. Results will be presented at national and international conferences, published in scientific journals and publicly disseminated to key stakeholders.

Keywords: Dementia, singing intervention, music, dance, non-pharmacological interventions, support services

Strengths and limitations of this study

- This study will assess the impact of two differing and unique singing interventions for people living with dementia (PwD) and their carers using a mixed methods approach and examine barriers and facilitators to assessing singing support services.
- A social return on investment analysis will be conducted to determine the social return for each singing support service.
- An important limitation of the study is that it does not include an independent control group.
- The inability to follow up after the 12-week intervention period means that the long-term effects of the interventions cannot be determined in this study.
- As participants cannot be blinded, self-report measures may include an element of bias in the responses.

For peer review only - http://bmjopen.bmj.com/site/about/guidelines.xhtml

INTRODUCTION

Dementia currently affects more than 55 million people worldwide [1]. Dementia has detrimental effects on cognitive functioning [2] and often leads to behavioural and psychological changes, including aggression, anxiety, hallucinations and culturally inappropriate behaviours [3-5]. Dementia has profound effects on people's quality of life and ability to perform everyday tasks [6-8]. Pharmacological interventions can aid in the management of cognitive symptoms for PwD [9]. However, the benefits are often short-lived and are limited for behavioural symptoms such as anxiety, agitation and depressive symptoms [10]. Pharmacological approaches to manage behavioural symptoms in PwD include antipsychotics, anxiolytics, hypnotics and antidepressant medications [11]. There are often negative side effects associated with using these medications including increased occurrence of strokes [12] and mortality [13,14]. Research has also suggested that antipsychotics may worsen quality of life and cognition [15-17]. The negative side effects of pharmacological interventions highlight's the need for non-pharmacological interventions to reduce and help manage behavioural symptoms of dementia [18].

As a result, there is a strong need for non-pharmacological interventions such as music therapy which has demonstrated strong benefits for PwD [19,20]. Music therapies have been shown to improve mood, regulate emotion and relieve stress in older adults [21] and in PwD [22-24]. Music therapy as a whole consists of many components and applications and one specific and promising form of music therapy is singing interventions. The act of singing combines language, music and instinctive human behaviour to enhance neurological stimulation [25]. Research has linked singing to improved memory performance [26] and increased cognitive functioning as it utilises and engages brain pathways other than in plain speech, which is beneficial for people with advanced stages of dementia [27,28]. Group singing interventions may also help to improve social interactions between PwD, promoting relaxation and reducing levels of agitation [29,30]. Singing interventions in particular have demonstrated value and effectiveness within the realm of music therapies.

It should be noted that there is mixed evidence within the literature with a systematic review of music therapy for PwD finding that music listening had a greater effect on PwD compared with singing interventions [19]. However, in recent years, research has focused on specific aspects of music therapy which result in the best outcomes for PwD [31-33]. A systematic review of multiple variations of music therapy (ranging from passive music

For peer review only - http://bmjopen.bmj.com/site/about/guidelines.xhtml

listening to active singing sessions) for PwD in residential care identified singing as an important aspect for change and improvements in mood and reductions in behavioural disturbances [34]. Further evidence from a randomised control trial suggests that singing interventions which actively engage participants may have greater effects on mood and quality of life when compared with passive music therapy [31,35]. These studies indicate that singing and active engagement in music sessions may be a key component for significant improvements in PwD. This variation in findings may be due to variations in dementia severity with some studies suggesting that passive music therapy may be more beneficial in more advanced stages of dementia and more active music therapy involving singing more beneficial in the early to moderate stages [36].

PwD can often experience a decrease in physical activity levels post-diagnosis which can lead to adverse effects on physical health such as reductions in strength, balance, mobility and increased risk of physical frailty. Increased physical activity has been associated with positive cognitive and physical outcomes in PwD [37] and a reduction in symptom progression. Review evidence has found that multidomain interventions may be more beneficial for PwD [38]. Research examining dancing sessions in PwD has found that dancing can increase playfulness and sociability in PwD [39], improve levels of agitation and cognitive function [40] and also improve physical performance such as balance and walking speed [41]. Interventions that combine physical aspects alongside cognitive stimulation may lead to increased benefits and a wider range of positive outcomes. Research has not examined the effects of combining both singing and dancing.

Research has found that people with limited social networks and reduced social engagement may be more at risk of developing dementia compared to those with wider and richer social networks [42]. It is thought that social activity and engagement may be a protective factor against cognitive decline by increasing cognitive reserve to better maintain cognitive functioning and performance [42]. To our knowledge, no evaluations have examined the benefits of singing interventions combined with an eating-together element which provides a sociable experience alongside the singing session. Existing literature often focuses on the singing element of these interventions [43], however, there are many social aspects of the sessions which lead to the benefits for PwD and their carers [44,45] that have previously been overlooked. The current project will investigate the impacts of two established charity-based services provided by The Brain Charity and the Lyrics and Lunch Charity for PwD and their carers.

For peer review only - http://bmjopen.bmj.com/site/about/guidelines.xhtml

Most PwD live at home where they are often cared for and supported by their friends and family [46]. Family carers are a strong factor leading to more positive outcomes for PwD and can reduce or delay the need for residential care [47]. It can be hugely rewarding and fulfilling caring for a family member living with dementia [48], however, it can also be challenging and have negative impacts on the carers' mental and physical health and quality of life [47]. Due to this, interventions such as music therapy have been developed to promote well-being among unpaid carers [49]. Singing interventions have been found to improve the relationship between PwD and their carers in addition to easing carer burden when attended together [50]. The challenges of dementia are often not limited to the PwD, with relatives, carers and friends often profoundly impacted [51]. Therefore, there is a need for services to offer support not only for PwD but also for their carers [52,53].

Multiple barriers may prevent PwD from accessing and continuing to engage with an intervention such as cost of travel, increased reliance on paid and unpaid carers and cost of service and care [54,55]. Studies to date have not examined the potential barriers to accessing singing intervention services and specifically how these may differ based on socioeconomic position and geographical location. Accessing services after a diagnosis can be difficult [56] so understanding the benefits of a specific form of support and potential barriers to accessing these services is important. The current study examines barriers and facilitators for accessing and continuing to engage with singing intervention services and the organisations that provide these services.

Remote delivery of singing intervention services may be a solution to some barriers to attending singing interventions such as travel or reliance on carers. During the COVID-19 pandemic, multiple dementia support groups were moved online and demonstrated the potential for singing interventions to be delivered remotely [57,58]. However, the feasibility, acceptability and whether remote delivery yields similar benefits as in-person groups has been questioned [57]. For people living with advanced stages of dementia barriers may prevent them from attending in-person groups and in these cases, remote delivery may be a method to improve access and reach of these services.

It is important to quantify the value of interventions, not just in economic terms, but also in terms of social value. Social Return on Investment (SROI) is a method of cost-benefit analysis that assigns monetary values to social outcomes that usually would not be accounted for in standard financial evaluations. SROI analysis can be used to determine an intervention's social value in relation to financial investment [59]. SROI analysis can be a useful tool for examining interventions and to inform policy-making relating to social support

For peer review only - http://bmjopen.bmj.com/site/about/guidelines.xhtml

investments [60,61]. In the current project, the SROI will be examined for both support services.

This 2-year project aims to examine the benefits of established charity-provided dementia singing groups for both people living with dementia and their unpaid carers using both quantitative and qualitative methods. For this, the following interlinked research questions will be addressed: (1) ”Do singing interventions combined with a social lunch have an impact on well-being in PwD and their carers?”; (2) "Do singing and dancing intervention services have an impact on well-being in PwD and their carers?”; (3) “What barriers are encountered when accessing singing intervention services and what prevents continued engagement?”; (4) “Could remote delivery of singing interventions improve access and what barriers may there be with remote delivery of singing interventions?”; (5) “What is the social return on investment of the singing support services examined in this project?”.

METHODS AND ANALYSIS

Study overview

This mixed-methods study consists of four interlinked work packages (WPs) examining the influence of singing services provided by two registered third-sector organisations, Lyrics and Lunch and The Brain Charity. The barriers that people encounter when accessing and staying engaged with singing interventions will be examined and whether these disproportionately affect people based on sociodemographic factors specifically SEP and location (urban vs rural location). The potential of remote delivery of singing services to help improve wider access will also be assessed. To do this, semi-structured interviews will be conducted with key stakeholders including PwD, unpaid carers, service providers (music therapists, workshop coordinators) and local facilitators (paid carers, care home managers). From this data, recommendations for future delivery of singing support services will be developed with a focus on improving access and continued engagement. A SROI will be conducted to examine the social impact of both support services. A systematic review is also being conducted on community-based singing interventions for people living with dementia and whether they improve mood and quality of life to further inform the work (Prospero ID: CRD42023395907).

Two singing (and dancing) interventions

For peer review only - http://bmjopen.bmj.com/site/about/guidelines.xhtml

Intervention 1: Lyrics and Lunch

Lyrics and Lunch is a charity based in Lancaster with the aim to serve people with dementia and their carers by providing a singing group and a nourishing lunch in a sociable community environment. The sessions take place weekly or biweekly and last approximately 2 hours with the first hour consisting of a sociable lunch followed by an hour of group singing.
For the singing session, attendees will be provided with a book with the lyrics to each song. Each session will start with the group singing an original song that allows each person to introduce themselves. The session leader will play the piano or guitar and facilitate the group to sing along. Familiar and well-known songs will be sung throughout the sessions such as "Happy Together" by The Turtles. Around halfway through the sessions, percussion instruments will be given out and used to accompany the around 2-3 songs. The session will often include rounds in which people will be split into smaller groups and each group will sing the same melody in tandem. Lyrics and Lunch is a non-denominational Church-based organisation and a short optional reflective spiritual component is included at the end, but the sessions are open to people with any religion or none. The service is open to PwD living within the community and their carers.

Intervention 2: The Brain Charity's Singing and Dancing Groups

The Brain Charity is based in Liverpool, and it supports people with neurological conditions. The charity runs singing (Music Makes Us Sing) and dancing (Music Makes Us Dance) sessions in both community and residential care home settings for PwD and their carers. The singing sessions last approximately one hour and involve engaging in communal singing of familiar songs such as "My Bonnie" by The Beatles led by two session leaders. Lyrics are presented on a TV screen and attendees are positioned in a semi-circle in view of the screen. The singing sessions have been designed alongside speech and language therapists and focus on speech, language and breathing exercises to improve speech, pitch and rhythm such as call and response and clapping rhythm exercises. Attendees are encouraged to sing and dance along to the songs by session facilitators. The dance sessions last approximately one hour and involve physically engaging chair-based dance moves designed alongside a physical therapist and also incorporates group singing. The sessions are run by two session facilitators who before each song will demonstrate simple dance routines to familiar songs such as "Hit the Road Jack" by Ray Charles. During the songs, session facilitators will give cues to the next dance moves so attendees can follow the dance routines. Props will be used throughout the

For peer review only - http://bmjopen.bmj.com/site/about/guidelines.xhtml

sessions such as scarves, percussion instruments and balloons. The sessions are run weekly in 12-week blocks and follow a consistent format each week using similar songs and dance routines so attendees can become familiar with them.

Recruitment and sample size

For intervention 1 (Lyrics and Lunch): PwD and carers who have accessed the Lyrics and Lunch groups or who are beginning to attend will be invited to participate. A power analysis was conducted using G*Power software version 3.1.9.7. For the analysis, the power level was set at .80 with an error of .05 [62]. The effect size (0.44) was based on a mean effect size taken across eight studies examining active music therapy and reported in Vasionytė et al [63] and based on the primary outcome measure of mood and depressive symptoms. Results revealed a minimum sample size of N=55 is necessary to achieve a power of .80 at an alpha of .05. Therefore, to account for participant dropout and retention rates, the project will aim to recruit around n=75 PwD and n=75 of their carers from the Lyrics and Lunch groups.

For intervention 2 (The Brain Charity): PwD and carers who have expressed interest in accessing the Brain Charity's singing and dancing groups will be recruited before attending the groups. Separate samples will be collected for the singing intervention group and the dancing intervention group. Participants will be recruited from multiple singing and dancing intervention groups across the Liverpool area. These groups will include PwD living within the community and people living in residential care homes. The singing groups and dancing groups will be run by the same session facilitators.

The power analysis remained consistence with intervention 1 and revealed a minimum sample size of N= 55 is necessary to achieve a power of .80 at an alpha of .05. Therefore, across both intervention groups a total sample size of N=300 will be recruited to account for participant drop out. This will consist of n=150 PwD (n=75 from the singing group and n=75 from the dancing group) and n=150 of their carers (n=75 from the singing group and n=75 from the dancing group). Data collection for the study commenced on the 25th of May 2023.

Work Package I – Understanding the impacts of the Lyrics and Lunch support service on people with dementia and their carers

WP1 will address the first research question by assessing the impact of a singing intervention service that includes a sociable lunch aspect on PwD and their carers. This study will use

For peer review only - http://bmjopen.bmj.com/site/about/guidelines.xhtml

quantitative methods to examine the impact of attending the singing service on cognition, social isolation, loneliness, quality of life, neuropsychiatric symptoms such as agitation or other responsive behaviours, mood (depression/anxiety) and carer burden. Additionally, the core outcome set of 13 outcomes of interventions valued by people living at home with dementia, developed directly with PwD, informal and professional carers [64] will be used. The 13 outcomes were classified into 4 domains including friendly neighbourhood and home, independence, self-managing dementia symptoms and quality of life and will be examined using a bespoke quantitative measure, an 18-item measure adapted from the Adults Social Care Outcome assessment [65], the engagement and independence in dementia questionnaire [66] and Older Americans' Resources and Services Instrumental Activities of Daily Living assessment [67]. Outcome measures will be examined when the sessions are running (ON session) and compared to periods when the session is not running such as summer holidays and Christmas breaks (OFF session) (Table 1). Participants will be required to have attended the Lyrics and Lunch sessions for a minimum period of 4 weeks before the ON session assessment. OFF-session assessments will be completed after the sessions have not been attended for a minimum of 4 weeks. The Lyrics and Lunch sessions run weekly or biweekly.

Table 1. Assessments to be completed by PwD and their carers

Assessment time period	Assessment completed by person living with dementia	Assessment completed by unpaid carer
**ON session (Participant has attended service for a minimum of 4 weeks)**	QIDS-SR, ACE, DemQOL, DSSI-10	QIDS-SR, DSSI-10, NPI-Q, DemQOL-Proxy, Zarit burden questionnaire
**OFF session (Participant has not attended a session for a minimum of 4 weeks)**	QIDS-SR, ACE, DemQOL, DSSI-10	QIDS-SR, DSSI-10, NPI-Q, DemQOL-Proxy, Zarit burden questionnaire

Measures

Standard outcome measures that will be assessed in PwD and their carers will include cognition assessed using a shortened version of the Addenbrooke's cognitive examination

For peer review only - http://bmjopen.bmj.com/site/about/guidelines.xhtml

(ACE-III) [68] where participant's verbal fluency and attention will be assessed, quality of life assessed using the dementia quality of life measure (DemQOL and DemQOL-Proxy) [69], social Isolation assessed using the Duke Social Support Index (DSSI-10) [70], loneliness assessed using Three-item loneliness UCLA Measure [71], mood and depressive symptoms assessed using the Quick Inventory of Depressive Symptomatology (QIDS-SR) [72], neuropsychiatric symptoms assessed using Neuropsychiatric Inventory Questionnaire (NPI-Q) completed by carers [73] and carer burden assessed using the Zarit burden questionnaire[74].

Demographic information will be collected from participants at the first assessment including age, gender, ethnicity, highest education level (an indicator of SEP), number of years in formal education (an indicator of SEP), years since diagnosis and years since symptom onset. Participant attendance will be recorded to determine adherence to the intervention. The Global Deterioration Scale for Assessment of Primary Degenerative Dementia will be completed by carers to determine dementia severity [75].

It is hypothesised that attendance to singing interventions will increase quality of life and mood and reduce depressive and neuropsychiatric symptoms in PwD and their carers. Social isolation can be prominent in PwD and attending the singing intervention may reduce feelings of social isolation. Due to this, it is hypothesised that attending the singing intervention will result in reduced self-reported feelings of social isolation and loneliness in both PwD and their carers. It is hypothesised that both PwD and their carers will show improvements in the core outcome measures during the times that they are attending the singing intervention compared to the period when they are not attending the sessions.

Data analysis

To examine whether there were changes in outcome variables during on and off periods and to assess whether singing intervention support services are associated with changes in mood/depressive symptoms, quality of life, cognition and social isolation in people living with dementia and their carers, linear regression models will be used with time (on vs off) as the exposure variable. For the model examining outcomes in people living with dementia, outcome variables will be the QIDS-SR score, ACE-III score, DemQOL score, DSSI-10 score, NPI-Q score and Core outcome set score. Covariates will include participant age, years since diagnosis, years since symptom onset and number of sessions attended. A separate

For peer review only - http://bmjopen.bmj.com/site/about/guidelines.xhtml

model will examine outcome measures in dementia carers, outcome measures will be DSSI-10 score, QIDS-SR score and carer burden.

Work Package II – Understanding the impacts of singing and dancing support service conducted by The Brain Charity on people with dementia and their carers

WP2 will address the second research question and the impacts of attending either a 12-week singing intervention or a 12-week dancing intervention that also involves some singing for PwD and their carers. Participants will only attend one intervention service (either the singing or the dancing intervention). The benefits of dementia singing and dancing groups for both PwD and their unpaid carers, both community-residing and those living in care homes (conducted within the care home), will be examined using a mixed-methods approach. The project will assess the influence of the services on cognition, mood, social isolation, loneliness, carer burden and neuropsychiatric symptoms.

Measures

The following outcome measures will be used: DemQol, DemQol-Proxy, QIDS-SR, ACE-III (shortened version assessing verbal fluency and attention only), NPI-Q, DSSI-10, and Zarit burden questionnaire (Figure 1). Outcome measures will be examined at baseline (before the start of the 12-week block of sessions) and then one week after the final session, thus providing pre and post-intervention measures. The QIDS-SR will be completed every four weeks during the 12 weeks to examine more subtle changes in mood and depressive symptoms over the 12 weeks (Figure 1). The dance elements included in the dancing sessions may have impacts on the physical health and frailty of PwD attending the sessions. Due to this, The Physical Frailty Phenotype [76] measure of physical frailty will be included for the dancing group that will assess weight loss, grip strength, exhaustion, gait speed and physical activity. The Physical Frailty Phenotype will be completed pre and post-intervention.

Similar to WP1, participant attendance will be recorded to determine adherence to the intervention. Demographic information will be collected at baseline, including age, gender, ethnicity, highest education level (an indicator of SEP), number of years in formal education (an indicator of SEP), years since diagnosis and years since symptom onset. The Global Deterioration Scale for Assessment of Primary Degenerative Dementia will be completed by carers to determine dementia severity [75].

For peer review only - http://bmjopen.bmj.com/site/about/guidelines.xhtml

Data analysis

Consistent with WP1, we will examine whether there were changes in outcome variables pre vs. post-intervention and assess whether singing and dancing interventions are associated with changes in mood/depressive symptoms, quality of life, cognition and social isolation in people living with dementia and their carers. Linear regression models will be used with time (pre/post) and intervention type (singing or dancing intervention) as the exposure variables. The outcome variables will be QIDS-SR score, ACE-III score, DemQOL score, DSSI-10 score, Zarit burden score, NPI-Q score, sit-to-stand assessment score and gait assessment score. Covariates will include participant age, years since diagnosis, years since symptom onset, ACE score (dementia severity), number of sessions attended and participant residence (community-dwelling or care home). Separate models will be conducted to examine the singing and dancing service's effect on people living with dementia and then their carers and for each of the outcome measures.

Work Package III – Understanding the acceptability, remote delivery and barriers to accessing and continued engagement with singing interventions

WP3 will address research questions 3 and 4 and will examine barriers to accessing and continued engagement with singing interventions and how these factors may differ based on SEP and location (urban vs rural location). Accessibility barriers are likely to vary across these areas and depend on people's SEP and should be examined and acknowledged when rolling out intervention programmes on a wider scale.

A series of semi-structured follow-up interviews will be conducted with a subset of PwD, unpaid carers, service providers (music therapists, workshop coordinators) and local facilitators (paid carers, care home managers). The topic guide(s) will be coproduced with the teams and four public advisers consisting of a person living with dementia, a paid carer, an unpaid carer and a music teacher (JM) who runs the sessions.

The first aim will focus on barriers to access and continued engagement with the intervention service. To address this, a subset of 20 people will be recruited (approx. 10 PwD and 10 unpaid carers) from both interventions, resulting in a total of 40 interviews. This initial sample size determination was guided by information power methods [77]. These interviews will focus on the following topics: acceptability of the services, impacts of the

For peer review only - http://bmjopen.bmj.com/site/about/guidelines.xhtml

service on mood and overall quality of life and accessibility including barriers to access and continued engagement.

Our second aim is to examine the potential of services to be adapted for remote delivery and potential barriers. When discussing remote access, adaptations made to the services when COVID-19 restrictions were in place will be discussed and how successful these remote access adaptations were. To address this, approximately 20 people will be recruited: 10 service providers (music therapists, workshop coordinators) and 10 local facilitators (paid carers, care home managers). These interviews will focus on the following topics: impacts of the service on PwD and their carers, and barriers to remote delivery of singing. Again, this initial sample size determination was guided by information power methods [77]. Preliminary analysis will be used to determine the power of the analysis during the data collection phase to determine if the sample size calculation is correct. After the first three interviews, a first review of the data will be conducted, and first suggestions of relevant theory/themes will be made. This assessment will be considered again before closing data collection. Data will be analysed using Braun & Clarke's reflexive thematic analysis [78], and public advisors will assist with coding the transcripts.

Work Package IV – Examination of the social impact of singing interventions

WP4 will address research question 5 and the social impact of singing interventions examined by conducting an evaluative social return on investment (SROI) analysis on both services. Using standardised methods and following the seven principles a SROI will be conducted [79]. Separate SROI analyses will be conducted for the Lyrics and Lunch Charity and The Brain Charity. The methods and procedure employed for both analyses will remain consistent. An impact map will be created to calculate the social value generated by the intervention service which involves six stages [80]. Stage 1 will identify key stakeholders and people likely to be impacted by the services such as PwD, relatives, unpaid and paid carers, Lyrics and Lunch and The Brain Charity. Stage 2 will use quantitative data collected for WPs I and II on the effects of the intervention on quality of life, mood, carer burden, depressive symptoms and agitation levels and will map identified outcomes creating an impact map. This stage will identify what changes occurred and for whom. Stage 3 will provide a monetised value to each of the outcomes including those that do not have a price attached to them such as changes in quality of life. For outcome measures that do not have a clear

For peer review only - http://bmjopen.bmj.com/site/about/guidelines.xhtml

monetary value, established SROI value banks such as Social Value UK will be used to identify suitable financial proxies [81]. Stage 4 will establish impact by accounting for attribution, deadweight, displacement and drop-off. These adjustments are made to ensure that social value is not overclaimed [79]. Percentage values will be determined for attribution which considers changes or outcomes declared in the analysis that may not be due to just the singing intervention but may be due to other support services. A percentage value for the deadweight will be determined which considers how much change in the outcome values would have happened regardless of the singing intervention programme. Deadweight will be calculated as a 10% reduction in overall value for every resource the participant identifies as an alternative to the singing support service, for example if participants attend other support groups. Stage 5 will calculate the SROI benefits by adding up and subtracting any negatives (attribution, distribution, drop-off and deadweight), and this will be compared to the investment cost of the service. This calculation will provide us with a SROI ratio which demonstrates the social value of the service in relation to the cost invested, for example, an SROI ratio of £3.50: £1 means that for every pound invested, there is £3.50 of social value created. Stage 6 will disseminate the findings with key stakeholders and recommendations created for future delivery of the service.

Patient and public involvement

This project has been developed and conceptualised alongside members of the public and key stakeholders. Our formal project team includes four public advisors including a person living with dementia (SM), two carers (one paid (SP) and one unpaid (HB)) and a music teacher (JM) who runs the singing sessions. All public advisors have previous experience and involvement with the singing sessions provided by The Brain Charity or the Lyrics and Lunch Charity. Public advisors contribute to all aspects of this research project, from designing study documents and advising on outcome measures to the interpretation of findings and dissemination of the research. Public advisors are reimbursed for the time they contribute to the project.

ETHICS AND DISSEMINATION

This project has received ethical approval from the University of Liverpool's Ethics Committee (App ref: 12374) and Lancaster University's Ethics Committee (App ref: 3442). All methods will be conducted in accordance with the relevant guidelines and regulations and

For peer review only - http://bmjopen.bmj.com/site/about/guidelines.xhtml

all participants will provide informed consent to participate. Findings from this project will be presented at national and international conferences and published in scientific journals. Findings will be publicly disseminated to key stakeholders and within the community and the organisations included in this study will use the outcomes of the research for their own continuous improvement strategies.

DISCUSSION

Using a mixed-methods design, this project seeks to explore the influence of two established singing intervention services for PwD and their carers. The study aims to collect a large sample of key stakeholders to assess the influence of attending singing support services on well-being and quality of life through a combination of quantitative and qualitative methods. The findings from this research will highlight the impact of singing support services for PwD and their carers and will allow the SROI of such services to be evaluated. The project will examine the barriers that people encounter when accessing and continuing to engage with singing interventions and whether these barriers disproportionately affect people from lower SEP. It can be challenging to access support services after a diagnosis of dementia [56] and exploring ways to make services more widely and easily accessible is important. Methods to reduce potential barriers to access such as travel, increased reliance on carers and availability of services will be explored alongside the potential for remote delivery of singing services to improve and expand access.

A limitation of this study that should be noted is the lack of an independent control group. This study is therefore unable to robustly examine the impact of each intervention. However, it will provide important information on the acceptability and potential impact of the support service for PwD and their careers and barriers to access or engagement. This study will provide an important knowledge base for future studies and provide evidence of whether larger randomised control trials are warranted. A further limitation is that as participants cannot be blinded, self-report measures may include an element of bias in the responses. Additionally, this study is unable to determine the long-term effects of the interventions and cannot determine whether any effects of the intervention persist or diminish after the 12-week intervention. Future research should include further follow-up assessments post-intervention to determine long-term effects.

If the results from the current study suggest that singing support services have a positive impact on PwD and their carers, then support services could be scaled up and applied to other regions and settings to improve access to beneficial support services. Examining the

For peer review only - http://bmjopen.bmj.com/site/about/guidelines.xhtml

SROI will provide insight into the cost-effectiveness of the support services and if they will be suitable to apply in lower-income areas and regions both nationally and internationally. The findings from this project will be used to develop recommendations for future delivery of singing intervention services.

Abbreviation list

PwD	People living with dementia
WP	Work package
SEP	Socioeconomic Position
DemQOL	Dementia quality of life measure
ACE-III	Addenbrooke's cognitive examination
DSSI-10	Duke Social Support Index
QIDS-SR	Quick Inventory of Depressive Symptomatology
NPI-Q	Neuropsychiatric Inventory Questionnaire
SROI	Social return on investment

Competing interests: Carol Holland, Jeanette Main and Steve Pendrill are trustees of the Lyrics and Lunch Charity. All other authors declare no conflict of interest.

Consent for publication: N/A.

Funding: National Institute for Health and Care Research Applied Research Collaboration North West Coast (ARC NWC) (Award/Grant no: NA) and The Alzheimer’s Society (Award/Grant no: NA).

Contributors: Conceptualisation and methodology design contributions and project administration contributions were made by all authors (MP, KH, KW, FA, HB, CH, HB, JM, SM, SP, CG). The original draft preparation contributions were made by MP. MP, KH, KW, FA, HB, CH contributed to revising the manuscript. All authors (MP, KH, KW, RA, HB, CH, HB, JM, SM, SP, CG) read and agreed to the published version of the manuscript.

Data availability statement: Data generated in this study will be made available on Open Science Framework upon completion of the study.

References

For peer review only - http://bmjopen.bmj.com/site/about/guidelines.xhtml

1. World Health Organization . Geneva, Switzerland: World Health Organization; 2021. Fact sheets of dementia [Internet]. Available from: https:// www.who.int/news-room/fact-sheets/detail/dementia
2. Sliwinski M, Lipton RB, Buschke H, Stewart W. The effects of preclinical dementia on estimates of normal cognitive functioning in aging. The Journals of Gerontology Series B: Psychological Sciences and Social Sciences. 1996 Jul 1;51(4):P217-25.
3. Srikanth S, Nagaraja AV, Ratnavalli E. Neuropsychiatric symptoms in dementia-frequency, relationship to dementia severity and comparison in Alzheimer's disease, vascular dementia and frontotemporal dementia. Journal of the neurological sciences. 2005 Sep 15;236(1-2):43-8.
4. Cagnin A, Gnoato F, Jelcic N, Favaretto S, Zarantonello G, Ermani M, Dam M. Clinical and cognitive correlates of visual hallucinations in dementia with Lewy bodies. Journal of Neurology, Neurosurgery & Psychiatry. 2013 May 1;84(5):505-10.
5. Kwak YT, Yang Y, Koo MS. Anxiety in dementia. Dementia and neurocognitive disorders. 2017 Jun;16(2):33.
6. Banerjee S, Smith SC, Lamping DL, Harwood RH, Foley B, Smith P, Murray J, Prince M, Levin E, Mann A, Knapp M. Quality of life in dementia: more than just cognition. An analysis of associations with quality of life in dementia. Journal of Neurology, Neurosurgery & Psychiatry. 2006 Feb 1;77(2):146-8.
7. Giebel CM, Sutcliffe C, Challis D. Hierarchical decline of the initiative and performance of complex activities of daily living in dementia. Journal of geriatric psychiatry and neurology. 2017;30(2):96-103.
8. Resnick B, Galik E, Kolanowski A, VanHaitsma K, Boltz M, Zhu S, Ellis J, Behrens L, Eshraghi K, Renn C, Dorsey SG. The relationship between pain, function, behavioral, and psychological symptoms of dementia and quality of life. Pain Management Nursing. 2022 Feb 1;23(1):55-61.
9. Watts KE, Storr NJ, Barr PG, Rajkumar AP. Systematic review of pharmacological interventions for people with Lewy body dementia. Aging & Mental Health. 2023 Feb 1;27(2):203-16.
10. Gerlach LB, Kales HC. Pharmacological management of neuropsychiatric symptoms of dementia. Current treatment options in psychiatry. 2020 Dec;7:489-507.
11. Gustafsson M, Karlsson S, Gustafson Y, Lövheim H. Psychotropic drug use among people with dementia–a six-month follow-up study. BMC Pharmacology and Toxicology. 2013 Dec;14(1):1-9.
12. Maidment ID, Damery S, Campbell N, Seare N, Fox C, Iliffe S, Hilton A, Brown G, Barnes N, Wilcock J, Randle E. Medication review plus person-centred care: a feasibility study of a pharmacy-health psychology dual intervention to improve care for people living with dementia. BMC psychiatry. 2018 Dec;18(1):1-1.
13. Maust DT, Kim HM, Seyfried LS, Chiang C, Kavanagh J, Schneider LS, Kales HC. Antipsychotics, other psychotropics, and the risk of death in patients with dementia: number needed to harm. JAMA psychiatry. 2015 May 1;72(5):438-45.
14. Tampi RR, Tampi DJ, Balachandran S, Srinivasan S. Antipsychotic use in dementia: a systematic review of benefits and risks from meta-analyses. Therapeutic advances in chronic disease. 2016 Sep;7(5):229-45.
15. Ballard C, Corbett A. Management of neuropsychiatric symptoms in people with dementia. CNS drugs. 2010 Sep;24:729-39.
16. Maidment ID, Shaw RL, Killick K, Damery S, Hilton A, Wilcock J, Barnes N, Brown G, Gillespie S, Fox C, Barton G. Improving the management of behaviour that challenges associated with dementia in care homes: protocol for pharmacy–health psychology intervention feasibility study. BMJ open. 2016 Mar 1;6(3):e010279.
17. Rochon PA, Normand SL, Gomes T, Gill SS, Anderson GM, Melo M, Sykora K, Lipscombe L, Bell CM, Gurwitz JH. Antipsychotic therapy and short-term serious events in older adults with dementia. Archives of internal medicine. 2008 May 26;168(10):1090-6.

For peer review only - http://bmjopen.bmj.com/site/about/guidelines.xhtml

18. O'Donnell E, Holland C, Swarbrick C. Strategies used by care home staff to manage behaviour that challenges in dementia: A systematic review of qualitative studies. International Journal of Nursing Studies. 2022 Apr 22:104260.
19. Moreno-Morales C, Calero R, Moreno-Morales P, Pintado C. Music therapy in the treatment of dementia: A systematic review and meta-analysis. Frontiers in medicine. 2020 May 19;7:160.
20. Dimitriou TD, Verykouki E, Papatriantafyllou J, Konsta A, Kazis D, Tsolaki M. Non-Pharmacological interventions for the anxiety in patients with dementia. A cross-over randomised controlled trial. Behavioural brain research. 2020 Jul 15;390:112617.
21. Clair AA, Memmott J. Therapeutic uses of music with older adults. American Music Therapy Association. 8455 Colesville Road Suite 1000, Silver Spring, MD 20910; 2008.
22. Davidson JW, Almeida RA. An exploratory study of the impact of group singing activities on lucidity, energy, focus, mood and relaxation for persons with dementia and their caregivers. Psychology of Well-Being. 2014 Dec;4(1):1-3.
23. Lee S, O'Neill D, Moss H. Promoting well-being among people with early-stage dementia and their family carers through community-based group singing: a phenomenological study. Arts & Health. 2022a Jan 2;14(1):85-101.
24. Lee S, Allison T, O'Neill D, Punch P, Helitzer E, Moss H. Integrative review of singing and music interventions for family carers of people living with dementia. Health Promotion International. 2022b May;37(Supplement_1):i49-61.
25. Jeffries, K. J., Fritz, J. B., & Braun, A. R. (2003). Words in melody: an H215O PET study of brain activation during singing and speaking. *Neuroreport*, *14*(5), 749-754.
26. Carruth EK. The effects of singing and the spaced retrieval technique on improving face-name recognition in nursing home residents with memory loss. Journal of Music Therapy. 1997 Oct 1;34(3):165-86.
27. Satoh M, Yuba T, Tabei KI, Okubo Y, Kida H, Sakuma H, Tomimoto H. Music therapy using singing training improves psychomotor speed in patients with Alzheimer's disease: a neuropsychological and fMRI study. Dementia and geriatric cognitive disorders extra. 2015;5(3):296-308.
28. Mansens D, Deeg DJ, Comijs HC. The association between singing and/or playing a musical instrument and cognitive functions in older adults. Aging & Mental Health. 2018 Aug 3;22(8):970-7.
29. Lin Y, Chu H, Yang CY, Chen CH, Chen SG, Chang HJ, Hsieh CJ, Chou KR. Effectiveness of group music intervention against agitated behavior in elderly persons with dementia. International journal of geriatric psychiatry. 2011 Jul;26(7):670-8.
30. Unadkat S, Camic PM, Vella-Burrows T. Understanding the experience of group singing for couples where one partner has a diagnosis of dementia. The Gerontologist. 2017 Jun 1;57(3):469-78.
31. Li HC, Wang HH, Chou FH, Chen KM. The effect of music therapy on cognitive functioning among older adults: A systematic review and meta-analysis. Journal of the American Medical Directors Association. 2015 Jan 1;16(1):71-7.
32. Lam HL, Li WT, Laher I, Wong RY. Effects of music therapy on patients with dementia—A systematic review. Geriatrics. 2020 Sep 25;5(4):62.
33. Dowson B, Schneider J. Online singing groups for people with dementia: scoping review. Public Health. 2021 May 1;194:196-201.
34. McDermott O, Crellin N, Ridder HM, Orrell M. Music therapy in dementia: a narrative synthesis systematic review. International journal of geriatric psychiatry. 2013 Aug;28(8):781-94.
35. Cho HK. The effects of music therapy-singing group on quality of life and affect of persons with dementia: a randomized controlled trial. Frontiers in medicine. 2018 Oct 15;5:279.
36. Baker FA, Lee YE, Sousa TV, Stretton-Smith PA, Tamplin J, Sveinsdottir V, Geretsegger M, Wake JD, Assmus J, Gold C. Clinical effectiveness of music interventions for dementia and depression in elderly care (MIDDEL): Australian cohort of an international pragmatic cluster-randomised controlled trial. The Lancet Healthy Longevity. 2022 Mar 1;3(3):e153-65.

For peer review only - http://bmjopen.bmj.com/site/about/guidelines.xhtml

37. Blankevoort CG, Van Heuvelen MJ, Boersma F, Luning H, De Jong J, Scherder EJ. Review of effects of physical activity on strength, balance, mobility and ADL performance in elderly subjects with dementia. Dementia and geriatric cognitive disorders. 2010 Oct 28;30(5):392-402.
38. Brasure M, Desai P, Davila H, Nelson VA, Calvert C, Jutkowitz E, Butler M, Fink HA, Ratner E, Hemmy LS, McCarten JR. Physical activity interventions in preventing cognitive decline and Alzheimer-type dementia: a systematic review. Annals of internal medicine. 2018 Jan 2;168(1):30-8.
39. Kontos P, Grigorovich A, Kosurko A, Bar RJ, Herron RV, Menec VH, Skinner MW. Dancing with dementia: Exploring the embodied dimensions of creativity and social engagement. The Gerontologist. 2021 Aug;61(5):714-23.
40. Salihu D, Wong EM, Bello UM, Kwan RY. Effects of dance intervention on agitation and cognitive functioning of people living with dementia in institutional care facilities: Systematic review. Geriatric Nursing. 2021 Nov 1;42(6):1332-40.
41. Bracco L, Poirier G, Pinto-Carral A, Mourey F. Effect of Dance Therapy on the Physical Abilities of Older Adults with Dementia: A Systematic Review. Eur J Geriatr Gerontol. 2021 Dec 1;3(3):134-42.
42. Fratiglioni L, Wang HX. Brain reserve hypothesis in dementia. Journal of Alzheimer's disease. 2007 Jan 1;12(1):11-22.
43. Adkins-Regan E. Activity dependent brain plasticity: does singing increase the volume of a song system nucleus? Theoretical comment on Sartor and Ball (2005).
44. Galinha I, Pinal D, Lima ML, Labisa-Palmeira A. The role of social and physiological variables on older adults' cognitive improvement after a group singing intervention: the Sing4health randomized controlled trial. Psychosocial Intervention. 2021;30(3):123-38.
45. Clark IN, Tamplin JD, Baker FA. Community-dwelling people living with dementia and their family caregivers experience enhanced relationships and feelings of well-being following therapeutic group singing: A qualitative thematic analysis. Frontiers in Psychology. 2018 Jul 30;9:1332.
46. Dickinson C, Dow J, Gibson G, Hayes L, Robalino S, Robinson L. Psychosocial intervention for carers of people with dementia: what components are most effective and when? A systematic review of systematic reviews. International psychogeriatrics. 2017 Jan;29(1):31-43.
47. Farina N, Page TE, Daley S, Brown A, Bowling A, Basset T, Livingston G, Knapp M, Murray J, Banerjee S. Factors associated with the quality of life of family carers of people with dementia: A systematic review. Alzheimer's & Dementia. 2017 May 1;13(5):572-81.
48. Laver K, Milte R, Dyer S, Crotty M. A systematic review and meta-analysis comparing carer focused and dyadic multicomponent interventions for carers of people with dementia. Journal of Aging and Health. 2017 Dec;29(8):1308-49.
49. Teahan A, Lafferty A, McAuliffe E, Phelan A, O'Sullivan L, O'Shea D, Nicholson E, Fealy G. Psychosocial interventions for family carers of people with dementia: A systematic review and meta-analysis. Journal of Aging and Health. 2020 Oct;32(9):1198-213.
50. Lee S, Allison T, O'Neill D, Punch P, Helitzer E, Moss H. Integrative review of singing and music interventions for family carers of people living with dementia. Health Promotion International. 2022 May;37(Supplement_1):i49-61.
51. Sutcliffe C, Giebel C, Bleijlevens M, Lethin C, Stolt M, Saks K, Soto ME, Meyer G, Zabalegui A, Chester H, Challis D. Caring for a person with dementia on the margins of long-term care: a perspective on burden from 8 European countries. Journal of the American Medical Directors Association. 2017 Nov 1;18(11):967-73.
52. Alwin J, Öberg B, Krevers B. Support/services among family caregivers of persons with dementia—perceived importance and services received. International Journal of Geriatric Psychiatry: A journal of the psychiatry of late life and allied sciences. 2010 Mar;25(3):240-8.
53. Bayly M, Morgan D, Chow AF, Kosteniuk J, Elliot V. Dementia-related education and support service availability, accessibility, and use in rural areas: Barriers and solutions. Canadian Journal on Aging/La Revue canadienne du vieillissement. 2020 Dec;39(4):545-85.

For peer review only - http://bmjopen.bmj.com/site/about/guidelines.xhtml

54. Morgan DG, Semchuk KM, Stewart NJ, D'arcy C. Rural families caring for a relative with dementia: Barriers to use of formal services. Social science & medicine. 2002 Oct 1;55(7):1129-42.
55. Hossain MZ, Khan HT. Barriers to access and ways to improve dementia services for a minority ethnic group in England. Journal of evaluation in clinical practice. 2020 Dec;26(6):1629-37.
56. Giebel C, Robertson S, Beaulen A, Zwakhalen S, Allen D, Verbeek H. "Nobody Seems to Know Where to Even Turn To": Barriers in Accessing and Utilising Dementia Care Services in England and The Netherlands. International Journal of Environmental Research and Public Health. 2021 Nov 22;18(22):12233.
57. Dowson B, Schneider J. Online singing groups for people with dementia: scoping review. Public Health. 2021 May 1;194:196-201.
58. Lee S, O'Neill D, Moss H. Dementia-inclusive group-singing online during COVID-19: A qualitative exploration. Nordic Journal of Music Therapy. 2022 Aug 8;31(4):308-26.
59. Hopkins G, Winrow E, Davies C, Seddon D. Beyond social prescribing—The use of social return on investment (SROI) analysis in integrated health and social care interventions in England and Wales: A protocol for a systematic review. Plos one. 2023 Feb 21;18(2):e0277386.
60. Jones C, Windle G, Edwards RT. Dementia and imagination: a social return on investment analysis framework for art activities for people living with dementia. The Gerontologist. 2020 Jan 24;60(1):112-23.
61. Bosco A, Schneider J, Broome E. The social value of the arts for care home residents in England: a social return on investment (SROI) analysis of the imagine arts programme. Maturitas. 2019 Jun 1;124:15-24.
62. Faul F, Erdfelder E, Lang AG, Buchner A. G* Power 3: A flexible statistical power analysis program for the social, behavioral, and biomedical sciences. Behavior research methods. 2007 May;39(2):175-91.
63. Vasionytė I, Madison G. Musical intervention for patients with dementia: a meta-analysis. Journal of clinical nursing. 2013 May;22(9-10):1203-16.
64. Reilly ST, Harding AJ, Morbey H, Ahmed F, Williamson PR, Swarbrick C, Leroi I, Davies L, Reeves D, Holland F, Hann M. What is important to people with dementia living at home? A set of core outcome items for use in the evaluation of non-pharmacological community-based health and social care interventions. Age and ageing. 2020 Jul 1;49(4):664-71.
65. Johnstone L, Page C. Using Adult Social Care Outcomes Toolkit (ASCOT) in the assessment and review process. Res Policy Plann. 2013;30(3):179-92.
66. Stoner, C.R., Orrell, M., & Spector, A. (2018). Psychometric properties and factor analysis of the Engagement and Independence in Dementia Questionnaire (EID-Q). Dementia and Geriatric Cognitive Disorders, 46, 3-4, 119 – 127. DOI:10.1159/000488484
67. Doble SE, Fisher AG. The dimensionality and validity of the Older Americans Resources and Services (OARS) Activities of Daily Living (ADL) scale. Journal of Outcome Measurement. 1998 Jan 1;2(1):4-24.
68. Hsieh S, Schubert S, Hoon C, Mioshi E, Hodges JR. Validation of the Addenbrooke's Cognitive Examination III in frontotemporal dementia and Alzheimer's disease. Dementia and geriatric cognitive disorders. 2013;36(3-4):242-50.
69. Smith SC, Lamping DL, Banerjee S, Harwood R, Foley B, Smith P, Cook JC, Murray J, Prince M, Levin E, Mann A. Measurement of health-related quality of life for people with dementia: development of a new instrument (DEMQOL) and an evaluation of current methodology. Health Technology Assessment (Winchester, England). 2005;9(10):1-iv.
70. Koenig HG, Westlund RE, George LK, Hughes DC, Blazer DG, Hybels C. Abbreviating the Duke Social Support Index for use in chronically ill elderly individuals. Psychosomatics. 1993 Jan 1;34(1):61-9.
71. Russell DW. UCLA Loneliness Scale (Version 3): Reliability, validity, and factor structure. Journal of personality assessment. 1996 Feb 1;66(1):20-40.

For peer review only - http://bmjopen.bmj.com/site/about/guidelines.xhtml

72. Rush AJ, Trivedi MH, Ibrahim HM, Carmody TJ, Arnow B, Klein DN, Markowitz JC, Ninan PT, Kornstein S, Manber R, Thase ME. The 16-Item Quick Inventory of Depressive Symptomatology (QIDS), clinician rating (QIDS-C), and self-report (QIDS-SR): a psychometric evaluation in patients with chronic major depression. Biological psychiatry. 2003 Sep 1;54(5):573-83.
73. Kaufer DI, Cummings JL, Ketchel P, Smith V, MacMillan A, Shelley T, Lopez OL, DeKosky ST. Validation of the NPI-Q, a brief clinical form of the Neuropsychiatric Inventory. The Journal of neuropsychiatry and clinical neurosciences. 2000 May;12(2):233-9.
74. Yap P. Validity and reliability of the Zarit Burden Interview in assessing caregiving burden. Ann Acad Med Singapore. 2010;39:758-63.0 May;12(2):233-9.
75. Reisberg B, Ferris SH, de Leon MJ, Crook T. Global Deterioration Scale. The American Journal of Psychiatry. 1983.
76. Fried LP, Tangen CM, Walston JD, Newman AB, Hirsch C, Gottdiener JS. E Seeman, T.; Tracy, RP; Kop, WJ; Burke, GL; et al. Frailty in older adults: Evidence for a phenotype. J. Gerontol. Ser. A Boil. Sci. Med. Sci. 2001;56:M146-57.
77. Malterud K, Siersma VD, Guassora AD. Sample size in qualitative interview studies: guided by information power. Qualitative health research. 2016 Nov;26(13):1753-60.
78. Braun, V., & Clarke, V. (2021). One size fits all? What counts as quality practice in (reflexive) thematic analysis?. *Qualitative research in psychology*, *18*(3), 328-352.
79. Fujiwara D. The seven principle problems of SROI. London: Simetrica Ltd. 2015 Aug.
80. Banke-Thomas AO, Madaj B, Charles A, van den Broek N. Social Return on Investment (SROI) methodology to account for value for money of public health interventions: a systematic review. BMC public health. 2015 Dec;15(1):1-4.
81. Social Value UK/HACT. SROI and HACT Social Value Bank Linkages Paper—Social Value UK. Social Value UK. 2015. [accessed 7 Oct 2022] Available from: https://socialvalueuk.org/resource/sroi-and-hact-social-value-bank-linkages-paper/

FIGURE TITLE

Figure 1. Study timeline

For peer review only - http://bmjopen.bmj.com/site/about/guidelines.xhtml

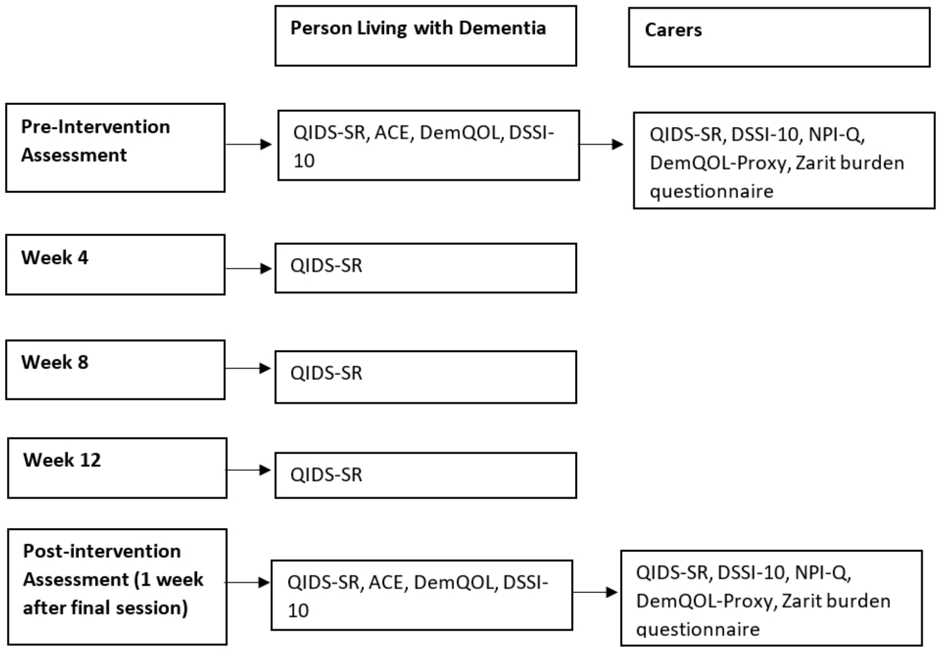

Figure 1. Timeline of cognitive assessments for PwD and unpaid carers

682x470mm (38 x 38 DPI)
